# Secondary Metabolites with Cytotoxic Activities from *Streptomyces* sp. BM-8 Isolated from the Feces of *Equus* *quagga*

**DOI:** 10.3390/molecules26247556

**Published:** 2021-12-13

**Authors:** Shengsheng Lu, Jianan Hu, Xi Xie, Runhong Zhou, Fangfang Li, Ruifeng Huang, Jian He

**Affiliations:** 1 Group of Peptides and Natural Products Research, School of Pharmaceutical Sciences, Southern Medical University, 1838 Guangzhou Avenue North, Guangzhou 510515, China; shengshenglu2016@163.com (S.L.); hjn0917@foxmail.com (J.H.); xixie2021@163.com (X.X.); zhourh2021@126.com (R.Z.); lff9282@163.com (F.L.); hrfeng1319@163.com (R.H.); 2 Department of Pharmacy, Affiliated Tumor Hospital of Guangxi Medical University, Nanning 530021, China

**Keywords:** animal intestinal bacteria, *Streptomyces* sp. BM-8, secondary metabolites, aliphatic acid, cytotoxic activities

## Abstract

A new aliphatic acid, compound **1**, together with six known metabolites, including nonactic acid (**2**), homononactic acid (**3**), ethyl homononactate (**4**), homononactylhomononactate (**5**), valinomycin (**6**), and cyclo-(Pro-Leu) (**7**), was isolated from the culture broth of *Streptomyces* sp. BM-8, an actinobacterial strain isolated from the feces of *Equus quagga*. The structures of these compounds were established by analyses of spectroscopic data, including 1D and 2D nuclear magnetic resonance spectra (NMR), as well as by HR-ESI-MS spectrometry and chemical derivative analyses. Additionally, a serial analogue of nonactic acid and homononacticacid (**8**–**21**) was synthesized. The cytotoxicity of **1**–**21** wastested against a panel of cancer cell lines, such as HT-29, MCF-7, A375 and K562, with MTT assay. In addition, the cytotoxicity tests revealed that **1** was less cytotoxic toward a panel of cancerous cells, as compared with valinomycin (**6**).

## 1. Introduction

Thus far, a large number of drugs/leads were derived from microbial secondary metabolites that were isolated from microbes living in different environments all around the world [1]. Among them, the intestinal microbiota has proven to be a new source of bioactive molecules that are used for the treatment of different kinds of diseases [2]. The host-microbe mutualism is a special ecosystem associated with the interactions between symbiotic bacteria and the hosts of plants or animals. As we have known, the intestinal microbiota arecomposed of a large number of heterogeneous commensal bacterial species that have coevolved with the host for very long periods of time. Therefore, it can be inferred that the metabolites produced by these microbials residing in the intestine might be safe and provide additional protection for their hosts’ homeostasis [3], and possess a strong potential for development as possible therapeutic agents [4], which has been supported by a lot of literature published in recent years. In addition, a large number of novel and bioactive metabolites associated with this special ecosystem have been identified [5,6,7].

In our previous study, a butanolide with potent anti-influenza A virus activity was isolated and identified from a bacterial strain of *Streptomyces* sp. SMU03 residing in an Asian elephant’s intestine via the bioassay-guided approach [8]. In our continuous search for bioactive compounds from intestinal bacteria, we identified an actinobacterium of *Streptomyces* sp. BM-8 from the feces of *Equus quagga*. Notably, the ethyl acetate extracts of the culture broth of this bacterium showed potent cytotoxic activity toward a panel of cancerous cell lines (with IC_50_ values in the low nanogram per milliliter range).

**Figure 1:**
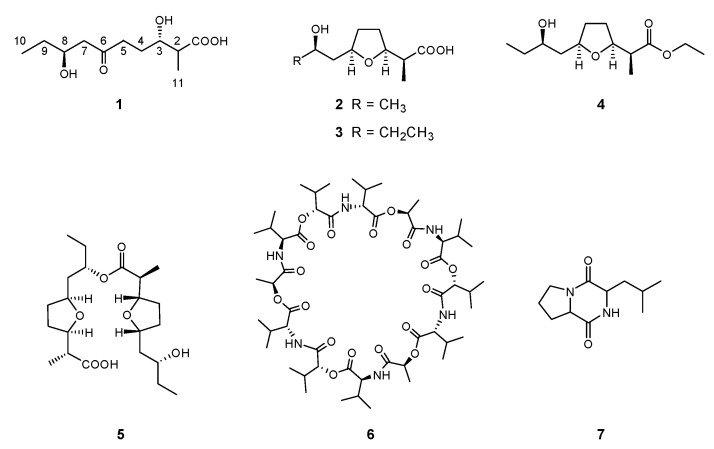
Structures of compounds **1**–**7**.

In this study, bioassay-guided isolation of the extraction of *Streptomyces* sp. BM-8resulted in the purification of a new compound (**1**) and six other known compounds, including nonactic acid (**2**) [9], homononactic acid (**3**) [9], ethyl homononactate (**4**) [10], homononactylhomononactate (**5**) [10], valinomycin (**6**) [11], cyclo-(Pro-Leu) (**7**) (Figure 1) [12], of which the chemical structures of **2**–**7** were established by comparison of NMR spectrum with literature (see Figures S8–S25). On the basis of this progress, 13 derivatives of nonactic acid were subsequently synthesized by a chemical approach to study the antitumor activity and structure–activity relationship of nonactic acid/homononactic acid. Herein, we report on the isolation, identification, and preliminary cytotoxic activities of these compounds.

## 2. Results

### 2.1. Structure Elucidation

Compound **1** was isolated as a colorless oil and was defined for the molecular formula of C_11_H_20_O_5_ based on high-resolution mass spectrometry analysis (electrospray ionization, [M − H]^−^ at *m/z* 231.1240, calculated as 231.1238; [M + Na]^+^ at *m/z* 255.1202, calculated as 255.1203; see Figures S6 and S7 in the Supporting Information), combined with data from ^1^H and ^13^C NMR spectra (see Table 1). The ^1^H NMR, ^13^C NMR, and DEPT spectra (in CDOD_3_) of 1 showed three methine protons from 2.29 to 3.98 ppm, including an aliphatic methine proton at *δ*_H_ 2.29 (quint, *J* = 6.16, 1 H), and two oxygenated methine protons at *δ*_H_ 3.49–3.54 (m, 1 H) and 3.92–3.98 (m, 1 H). The ^1^H NMR spectrum also displayed eight methylene protons at *δ*_H_ 2.62–2.65 (m, 2 H), 2.56 (d, *J* = 6.36, 2 H), 1.79–1.86 (m, 1 H), 1.62–1.71 (m, 1 H), 1.41–1.51 (m, 2 H), and two methyl protons at *δ*_H_ 1.18 (d, *J* = 7.04, 3 H), and 0.94 (t, *J* = 7.40, 3 H). The ^13^C NMR (see Table 1), HMQC, and HMBC spectral data displayed one carbonyl carbon at *δ*_c_ 212.9 ppm, one carboxyl carbon at *δ*_c_ 184.5 ppm, two oxygen-bearing carbons at *δ*_c_ 74.6 and 70.4 ppm, five aliphatic carbon signals at *δ*_c_ 50.92, 48.35, 30.35, 31.29, and 41.04 ppm, and two methyl carbon signals at *δ*_c_ 15.9 and 10.3 ppm. Considering the amount of the carbonyl carbon and the carboxyl carbon account for two unsaturation equivalents, it can be deduced that **1** possesses no ring system in the structure.

**Figure 2:**
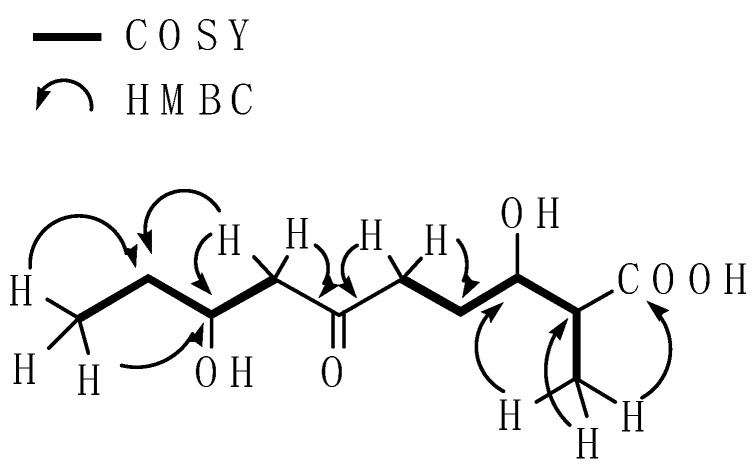
Key COSY and HMBC correlation of **1**.

The structure of **1** was therefore determined based on 2D NMR (HMQC, HMBC, and ^1^H-^1^HCOSY) correlations (Table 1 and Figure 2). Analysis of the^1^H-^1^H COSY spectroscopic data of **1** clearly showed that the connectivity of two substructures: the first aliphatic chain connected between C11 and C2 to C5 and the second chain between C7 to C10. The HMBC spectrum indicated correlations between H-11 (*δ*_H_ 1.18) and C-2 (*δ*_c_ 48.3), C-3 (*δ*_c_ 74.6), and the carboxyl (*δ*_c_ 184.5), suggesting that a carboxylic acid moiety was linked to C-2. The HMBC spectrum also showed a correlation between H-10 (*δ*_H_ 0.94) to C-8 (*δ*_c_ 70.4) and C-9 (*δ*_c_ 31.2), H-9 (*δ*_H_ 1.46) to C-7 (*δ*_c_ 50.9), C-8 (*δ*_c_ 70.4) and C-10 (*δ*_c_ 15.9), H-7 (*δ*_H_ 2.56) to C-8 (*δ*_c_ 70.4) and C-9 (*δ*_c_ 31.2), also indicating the connectivity between C-7, C-8, C-9, and C-10. The connectivity between H-5 (*δ*_H_ 2.63) to C-4 (*δ*_c_ 30.3) and C-6 (*δ*_c_ 212.9), H-7 (*δ*_H_ 2.56) to C-8 (*δ*_c_ 70.4) and C-6 (*δ*_c_ 212.9) was clearly observed in the HMBC spectrum, suggesting that the two aliphatic chains were linked by the carbonyl carbon (*δ*_c_ 212.9). These data established the planar structure of **1**, as shown in Figure 2.

**Figure 3:**
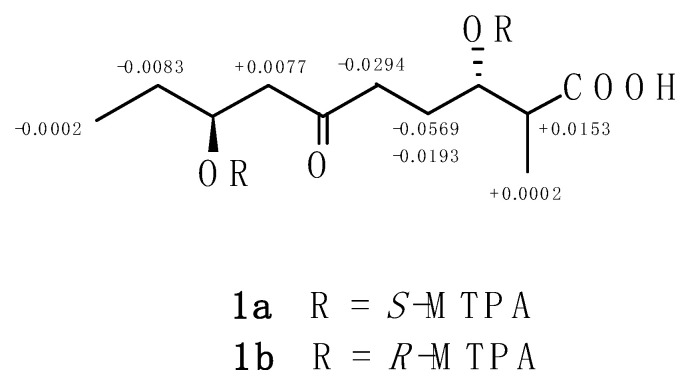
Δ*δ* values (Δ*δ* = *δ_S_* − *δ_R_*) around C-3 and C-8 obtained from **1a** and **1b**.

To determine the absolute configuration at C-3 and C-8 positions, a modified Mosher’s method was employed. First, *S*- and *R*-α-methoxy-α-(trifluoromethyl) phenylacetic acid (MTPA) was, respectively, reacting with anhydrous thionyl chloride (SOCl_2_) to yield *R*- and *S*-MTPA-Cl, which were then stirred with **1** and catalyzed by N,N-diisopropylethylamine (DIEA) to produce *S*- and *R*-MTPA ester (**1a** and **1b**). Calculation of the signals of the ^1^H NMR Δ*δ* values (Δ*δ* = *δ_S_* − *δ_R_*) established the steric structure of both C-3 and C-8 as *S* configurations (Figure 3).

Six previously reported compounds, nonactic acid (**2**), homononactic acid (**3**), ethyl homononactate (**4**), homononactyl homononactate (**5**), valinomycin (**6**), cyclo-(Pro-Leu) (**7**), were also isolated from the combined mycelial and culture broth extract of *Streptomyces* sp. BM8. The structures of **2**–**7** were established by comparison to previously reported spectroscopic data. And the ^1^H NMR and ^13^C NMR of **2**–**5** were listed in Table 2 and Table 3.

### 2.2. Synthesis of Serial Compounds Based on **2** and **3**

**Figure 4:**
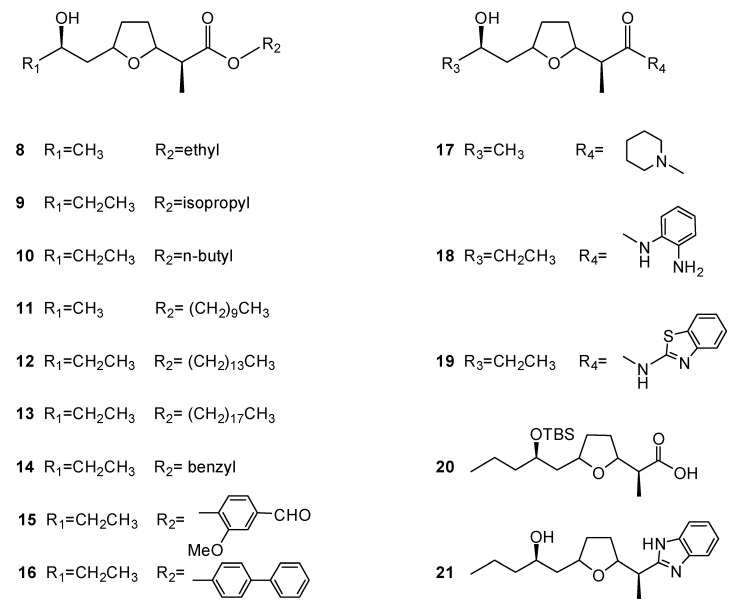
Structures of compounds **8**–**21**.

Since the main components of the culture broth of *Streptomyces* sp. BM8 were found to be known natural products nonactic acid (**2**) and homononactic acid (**3**), which showed different cytotoxicity against a panel of cancer cell lines, as indicated in Table 4, synthesis was employed for serial compounds **8**–**21**, as shown in Figure 4. Nonactic acid thatwas isolated from BM8 was reacted with appropriate derivatives, which led to the generation of compounds **8**, **11**, and **17,** while the synthesis of homononactic acid resulted in the preparation of the analogues **9**–**10**, **12**–**16**, and **18**–**21**. The cytotoxic activityof these newly synthesized analogues against different cancer cell lines was further evaluated.

### 2.3. Cytotoxicity

Compounds **1**–**21** were tested for their cytotoxicity against a panel of cell lines, including human colon cancer cell line HT-29, human breast cancer cell line MCF-7, human malignant melanoma cell line A375 and human chronic myeloid leukemia cell line K562. In addition, the human normal colon epithelial cell line NCM460 was also tested for comparison. As a result, **6** showed the most potent cytotoxicities toward all cell lines with the IC_50_ values ranging from 0.04 ± 0.01 µM to 1.66 ± 0.19 µM, while others were less active under the conditions tested (Table 4).

The synthesized analogues of nonactic acid or homononactic acid (**8**–**21**) were also evaluated for their in vitro cytotoxicity against these cell lines by the MTT assay with paclitaxel as apositive control. As shown in Table 4, the saturated fatty alcoholicesters of nonactic acid or homononactic acid, **8**–**13**, exhibited moderate cytotoxic activities with IC_50_ values in the micromolar range. Although **11** showed a slightly higher inhibitory effect toward the tested cells with IC_50_ ranging from 24.29 ± 0.03 μM to 44.21 ± 0.64 μM, structure–activity relationship could not be clearly observed since the cytotoxicities of **8**–**13** did not increase as the side chains of these compounds becoming longer. Replacing the fatty alcohol moiety with benzyl (**14**) led to average IC_50_ values ranging from 21.05 ± 3.66 μM to 89.92 ± 1.09 μM, suggesting that phenylesters of homononactic acid may have higher cytotoxicities. However, when the carboxyl group of the homononactic acid reacted with vanillin (**15**), 4-biphenylmethanol (**16**), pyridine (**17**), O-phenylenediamine (**18**), 2-Benzothiazolamine (**19**), the reaction products (**15**–**19**) showed different cytotoxic activities without an obvious structure–activity relationship, as did silylether (**20**) and the benzimidazole derivative (**21**). Furthermore, higher toxicity of most of the synthesized compounds towards the nontumorigenic cell line (NCM460) was observed in the MTT assay, as shown in Table 4, indicating that these compounds, including **9**, **14**, **18**, **20**, and **21,** may possess broad toxicity toward the cell lines tested.

## 3. Materials and Methods

### 3.1. General Experimental Procedures

One-dimensional and two-dimensional NMR spectra were recorded in deuterated chloroform or methanol-*d_4_* solution on a Bruker DRX-400 spectrometer (400 and 100 MHz for ^1^H and ^13^C NMR, respectively), and chemical shifts were referenced to the corresponding residual solvent signals (CD_3_OD, *δ*_H_ 3.31 ppm, *δ*_C_ 49.15 ppm; CDCl_3_, *δ*_H_ 7.26 ppm, *δ*_C_ 77.23 ppm). Optical rotations were recorded on an MCP500 automatic polarimeter (Anton Paar) with MeOH as solvent. High-resolution electrospray ionization (ESI) mass spectra were acquired on an Orbitrap Fusion^TM^Tribrid^TM^ spectrometer (Thermo Fisher Scientific, Waltham, MA, USA). HPLC analyses were measured using a Shimadzu SPD-M20A system equipped with a PDA detector and an Angela ODS column (4.6 × 150 mm, 5 μm). Silica gel (Qingdao Marine Chemical Factory, Qingdao, China), Sephadex LH-20 (Pharmacia) and C18 (40–63 μm, Merck) were used for column chromatography. Additionally, MTT experiments were performed with a Thermo Scientific Microplate Reader (Multiscan FC). Fetal bovine serum (FBS), RPMI1640 medium and Dulbecco’s modified Eagle medium (DMEM) were purchased from Thermo Fisher biochemical products (Bejing, China) Co., Ltd. Additionally, 3-(4,5-dimethylthiazol-2-yl)-3,5-dipheryltetrazoliu bromide (MTT) was obtained from Sigma-Aldrich (Shanghai, China) trading Co., Ltd.

### 3.2. Collection and Phylogenetic Analysis of Strain BM-8

The animal-derived bacterial strain BM8 was isolated from a feces sample excreted by adult *Equus quagga* collected in Guangzhou Zoo, Guangdong Province, China. Based on its 16S rRNA sequence (GenBank accession number MT912570) and morphology characteristics, the strain BM-8 was determined to be *Streptomyces* sp. by Guangzhou IGE biotechnology LID. A voucher specimen is preserved and available from the collection (No. BM-8) at Group of peptides and natural products research, School of Pharmaceutical Sciences, Southern Medical University in Guangzhou (510515), China.

### 3.3. Cultivation and Extraction

Bacterium BM-8 was cultivated in a 250 mL Erlenmeyer flask containing 50 mL of seed medium and shaken on a rotary shaker at 200 rpm at 28 °C for 2 days. Then, the seed medium was transferred to a 2 L Erlenmeyer flask containing 0.5 L of culture medium and cultivated for another 7 days at 200 rpm at 28 °C. The seed medium was composed of glucose 10 g/L, beef extract 3 g/L, peptone 3 g/L, soluble starch 20 g/L, yeast extract 5 g/L, and CaCO_3_ 3 g/L, adjusted to pH 7.0 by 1 M NaOH solution. The culture medium contained mannitol 30 g/L, glucose 10 g/L, yeast extract 5 g/L, ammonium succinate 1 g/L, K_2_HPO_4_ 0.5 g/L, MgSO_4_·7H_2_O 0.5 g/L, and 1 mL/L of trace element concentrate, and adjusted to pH 7.5 by 1 M NaOH solution. The trace element concentrate was prepared with FeSO_4_·7H_2_O 0.2 g, MnCl_2_·2H_2_O 0.1 g, and ZnSO_4_·7H_2_O 0.1 g dissolved in 100 mL of deionized water. The seed medium and culture medium were autoclaved before use.

After seven days of cultivation, the culture broth was transferred to a 50 mL test tube and centrifuged at 8000 rpm for 6 min. A total culture broth of 22.5 L was collected. The supernatant was subjected to a D-101 resin column, washed with deionized water until the effluent became colorless, and then eluted with ethanol to generate the crude extract (20 g) after being concentrated under reduced pressure. Then the crude extract was partitioned with CH_2_Cl_2_ (2.4 g) and EtOAc (1.6 g). The mycelium was extracted with ethanol three times to give 6.6 g of brown crude extract, which was partitioned with CH_2_Cl_2_ (1.2 g) and EtOAc (1.4 g).

### 3.4. Isolation and Purification

The combined mycelial and culture broth extract (6.6 g) was fractionated by open column chromatography on silica gel (40 g) eluting with a stepwise gradient of petroleum ether and ethyl acetate (15:1–0:1) and then dichloromethane and methanol (9:1–1:1) to yield eleven fractions (Fr. 1–Fr. 11). Fraction 3 (294 mg) was subjected to a Sephadex LH-20 column (250 g, 2.5 cm × 80 cm) and eluted with methanol at the flow rate of 0.8 mL/min gives two parts. Fraction 3-I (120 mg) was dried under vacuum and then chromatographed using a C18 column (2 cm × 35 cm) eluted with 80% MeOH-H_2_O, followed by preparative ODS column (90% MeOH-H_2_O, C18 Column, 20 mm × 250 mm, 5 μm) to afford compound **6** (33 mg). Fraction 3-II (32 mg) was purified by silica gel chromatography (column: 1 cm × 35 mm, petroleum ether:ethyl acetate 5:1~3:1), and C18 column (1 cm × 35 cm) eluted with 60~80% MeOH-H_2_O to give compound **4** (1.88 mg). Fraction **4** (77 mg) was first subjected to a Sephadex LH-2 (MeOH; flow rate, 0.8 mL/min), followed by a semi-preparative HPLC to yield compound **5** (11 mg). Fraction 5 (311 mg) was subjected to Sephadex LH-20 column (MeOH), then the major fraction (128 mg) was loaded onto a C18 column (2 cm × 35 cm) washed with 30% aqueous MeOH to afford a subfraction 5-I (64 mg), which was further purified by using by a semi-preparative HPLC to yield **3** (11 mg). Fraction 8 (299 mg) was subjected to Sephadex LH-20 column (MeOH) to afford a subfraction 8-I (260 mg), which was further purified on the C18 column (2 cm × 35 cm, MeOH-H_2_O, 40:60) and on a semi-preparative HPLC to yield compound **2** (17 mg). Similarly, fraction 10 (381 mg) was first subjected to a Sephadex LH-20 column (MeOH) to give a subfraction 10-I (93 mg), which was further chromatographed using a C18 column (1 cm × 35 cm, MeOH-H_2_O, 20:80) to afford compound **1** (2.3 mg) and compound **7** (27 mg).

Compound (**1**): colorless oil. ^1^H and ^13^C NMR data, see Table 1 and Figures S1–S5. [α]${}_{D}^{20}$ = −117.6° (*c* = 0.142 in MeOH). HR-ESI-MS: *m/z* 255.1202 [M + Na]^+^ (calcd for C_11_H_20_O_5_Na, 255.1203), *m/z* 231.1240 [M − H]^−^ (calcd for C_11_H_19_O_5_, 231.1238) (Figures S6 and S7).

Nonactic acid (**2**): colorless oil. ^1^H and ^13^C NMR data, see Table 2 and Table 3, Figures S8 and S9. ESI-MS (positive ion) *m/z* = 225.7 [M + Na]^+^ (Figure S10).

Homononactic acid (**3**): colorless oil. ^1^H and ^13^C NMR data, see Table 2 and Table 3 and Figures S11 and S12. ESI-MS (negative ion) *m/z* = 215.7 [M − H]^−^ (Figure S13).

Ethyl homononactate (**4**): colorless oil. ^1^H and ^13^C NMR data, see Table 2 and Table 3, Figures S14 and S15. ESI-MS (positive ion) *m/z* = 267.4 [M + Na]^+^ (Figure S16).

Homononactylhomonanactate (**5**): colorless oil. ^1^H and ^13^C NMR data, see Table 2 and Table 3, Figures S17 and S18. ESI-MS (positive ion) *m/z* = 415.6 [M + H]^+^, 437.5 [M + Na]^+^ (Figure S19).

Valinomycin (**6**): white solid. ^1^H and ^13^C NMR data, see Figures S20 and S21. ESI-MS (positive ion) *m/z* = 1128.5 [M + NH_3_]^+^ (Figure S22).

Cyclo-(Pro-Leu) (**7**): white solid. ^1^H and ^13^C NMR data, see Figures S23 and S24. ESI-MS (positive ion) *m/z* = 233.6 [M + Na]^+^ (Figure S25).

### 3.5. Determination of the Absolute Configuration at C-3 and C-8 by Modified Mosher’s Method

(*S*)-α-methoxy-α-(trifluoromethyl)phenylacetic acid ((*S*)-MTPA 1.3 mg) was added into 5 mL vial and dissolved in 200 μL of anhydrous thionyl chloride (SOCl_2_) [13].The mixtures were stirred for 4 h at room temperature and then dried under vacuum for about 5 min. Compound **1** (0.5 mg) dissolved in chloroform was added into the vial containing the mixture immediately. Then, 1 μL of N,N-diisopropylethylamine (DIEA) was added into the reaction mixtures and stirred for another 36 h. The crude product mixtures were dried under vacuum and purified by preparative TLC using a solvent system of 60% petroleum ether/ethyl acetate to give bis-S-MTPA ester (**1a**) (0.2 mg, R*f* = 0.55). (R)-MTPA (1.7 mg) was prepared with the same procedures and reacted with **1** (0.5 mg) stirring for 24 h. Additionally, bis-R-MTPA ester (**1b**) (0.6 mg, R*f* = 0.55) was yielded by preparative TLC.

### 3.6. General Procedures for Preparation of Compounds ***8***–***21***

General procedure for compounds **8**–**16**: A solution of nonactic/homononactic acid (4.3 mg) and vanillin (6.5 mg) in DCM/DMF (100 μL) was treated with 4-DMAP (1 mg, 0.008 mmol), and the mixture was cooled at 0 °C in an ice bath. Dicyclohexylcarbodiimide (20 mg) dissolved in 100 μL DCM was added dropwise to the above solution. The reaction mixture was stirred at room temperature overnight. After the end of the reaction (monitoring by thin-layer chromatography), the resulting solution was quenched with water (2 mL) and extracted with ethyl acetate (2 mL×3). The combined organic layers were dried under vacuum and purified by column chromatography on silica gel.

**8**: (89%); White solid; R*f* = 0.33 (PE/EA 2:1); ^1^H NMR (400 MHz, CDCl_3_) *δ*_H_(ppm): 4.22–4.07 (m, 3 H), 4.06–3.92 (m, 2 H), 2.56–2.46 (quint, 1 H), 1.27–1.24 (t, *J* = 7.2 Hz, 3 H), 1.21–1.19 (d, *J* = 6.3 Hz, 3 H), 1.13–1.11 (d, *J* = 7.1 Hz, 3 H). ^13^C NMR (100 MHz, CDCl_3_) *δ*: 175.06, 81.34, 77.40, 65.41, 60.71, 45.59, 42.89, 30.78, 29.00, 23.36, 14.42, 13.70.

**9**: (88%); White solid; R*f* = 0.70 (PE/EA 5:3); ^1^H NMR (400 MHz, CDCl_3_) *δ*_H_(ppm): 5.30 (s, 1 H), 5.06–4.99 (m, 1 H), 4.17–4.11 (m, 1 H), 4.00–3.95 (q, *J* = 6.9 Hz, 1 H), 3.76–3.70 (m, 1 H), 3.51–3.44 (m, 1 H), 2.52–2.45 (m, 1 H), 1.98 (m, 2 H), 1.72–1.67 (m, 2 H), 1.54–1.44 (m, 2 H), 1.25–1.22 (m,6H), 1.11–1.09 (m, 2 H), 0.95–0.91 (t, 3 H). ^13^C NMR (100 MHz, CDCl_3_) *δ*: 174.61, 81.34, 77.43, 70.76, 67.96, 45.70, 40.83, 30.88, 30.13, 28.93, 21.99, 21.96, 13.59, 10.39. ESI-MS: calcd for C_14_H_26_O_4_ [M + Na]^+^, 281.17; found, 281.03.

**10**: (94%); White solid; R*f* = 0.70 (PE/EA 5:3); ^1^H NMR (400 MHz, CDCl_3_) *δ*_H_(ppm): 4.17–4.12 (m, 1 H), 4.11–4.08 (t, *J* = 6.6 Hz, 2 H), 3.99 (q, *J* = 7.0, 6.4 Hz, 1 H), 3.77–3.68 (m, 1 H), 2.53 (quint, 1 H), 2.02–1.95 (m, 2 H), 1.72–1.67 (m, 2 H), 1.65–1.56 (m, 1 H), 1.54–1.44 (m, 2 H), 1.42–1.35 (m, 2 H), 1.13–1.11 (d, *J* = 7.0 Hz, 3 H), 0.98–0.70(m, 6 H). ^13^C NMR (100 MHz, CDCl_3_) *δ*: 175.11, 81.20, 77.44, 70.68, 64.59, 45.58, 40.94, 30.89, 30.22, 28.92, 19.32, 13.92, 13.64, 10.34. ESI-MS: calcd for C_15_H_28_O_4_ [M + Na]^+^, 295.19; found, 295.04.

**11**: (72%); Colorless oil; R*f* = 0.45 (PE/EA 4:1); ^1^H NMR (400 MHz, CDCl_3_) *δ*_H_(ppm): 4.2–3.8 (m, 5 H), 2.57–2.48 (quint, 1 H), 2.08–1.86 (m, 4 H), 1.74–1.57 (m, 6 H), 1.34–1.22 (m, 13 H), 1.21–1.18 (d, *J* = 6.3 Hz, 3 H), 1.12–1.11 (d, *J* = 7.0 Hz, 3 H), 0.88 (t, *J* = 6.7 Hz, 3 H). ^13^C NMR (100 MHz, CDCl_3_) *δ*: 175.08, 81.21, 77.43, 77.37, 65.41, 64.91, 45.57, 43.01, 32.10, 30.83, 29.75, 29.51, 29.46, 28.89, 28.83, 26.08, 23.43, 22.89, 14.32, 13.64. ESI-MS: calcd for C_20_H_38_O_4_ [M + Na]^+^, 365.27; found, 365.16.

**12**: (52%); White solid; R*f* = 0.25 (PE/EA 6.5:1); ^1^H NMR (400 MHz, CDCl_3_) *δ*_H_(ppm): 4.19–4.11 (m, 1 H), 4.10–4.07 (t, *J* = 6.8 Hz, 2 H), 4.02–3.97 (q, 6.7 Hz, 1 H), 3.77–3.69 (m, 1 H), 2.57–2.50 (m, 1 H), 2.00–1.97(t, *J* =6.8 Hz, 3 H), 1.77–1.57 (m, 7 H), 1.50 (tt, *J* = 13.1, 6.9 Hz, 2 H), 1.38–1.19 (m, 24 H), 1.12 (d, *J* = 7.0 Hz, 3 H), 0.93 (t, *J* = 7.4 Hz, 3 H), 0.88 (t, *J* = 6.7 Hz, 3 H). ^13^C NMR (100 MHz, CDCl_3_) *δ*: 175.09, 81.19, 77.43, 70.66, 64.91, 45.56, 40.89, 32.14, 30.87, 30.22, 29.91, 29.87, 29.81, 29.75, 29.57, 29.48, 28.91, 28.83, 26.09, 22.91, 14.33, 13.63, 10.36. ESI-MS: calcd for C_25_H_48_O_4_ [M + Na]^+^, 435.35; found, 435.18.

**13**: (50%); White solid; R*f* = 0.45 (PE/EA 4:1); ^1^H NMR (400 MHz, CDCl_3_) *δ*_H_(ppm): 4.16–4.11 (m, 1 H), 4.08 (t, *J* = 6.8 Hz, 2 H), 4.02–3.97 (m, 1 H), 3.73 (d, *J* = 7.7 Hz, 1 H), 2.57–2.50 (quint, 1 H), 2.00–1.97 (m, 2 H), 1.73–1.66 (m, 4 H), 1.65–1.57 (m, 4 H), 1.54–1.46 (m, 2 H), 1.27–1.17 (m, 27 H), 1.13–1.11 (d, *J* = 7.0 Hz, 3 H), 0.93 (t, *J* = 7.5 Hz, 3 H), 0.90–0.86 (t, 3 H). ^13^C NMR (100 MHz, CDCl_3_) *δ*: 175.09, 81.19, 77.44, 70.67, 64.91, 45.56, 40.89, 32.15, 31.66, 30.88, 30.54, 30.42, 30.23, 29.92, 29.88, 29.82, 29.76, 29.58, 29.49, 28.91, 28.84, 26.10, 22.91, 14.34, 13.63, 10.36. ESI-MS: calcd for C_29_H_56_O_4_ [M + Na]^+^, 491.41; found, 491.26.

**14**: (88%); Colorless oil; R*f* = 0.50 (PE/EA 5:2); ^1^H NMR (400 MHz, CDCl_3_) *δ*_H_(ppm): 7.36 (m, 5 H), 5.30 (s, 1 H), 5.14 (d, *J* = 3.2 Hz, 2 H), 4.13 (m, 1 H), 4.06–4.00 (m, 1 H), 3.74–3.68 (m, 1 H), 2.63–2.56 (quint, 1 H), 2.01–1.90 (m, 4 H), 1.70–1.66 (m, 2 H), 1.65–1.59 (m, 2 H), 1.15–1.13 (d, *J* =7.1 Hz, 3 H), 0.94–0.92 (t, *J* = 7.4 Hz, 3 H). ^13^C NMR (100 MHz, CDCl_3_) *δ*: 174.80, 136.31, 128.69, 128.34, 128.30, 81.08, 77.43, 77.40, 70.61, 66.44, 45.59, 41.09, 30.92, 30.27, 28.89, 13.65, 10.30. ESI-MS: calcd for C_18_H_26_O_4_ [M + Na]^+^, 329.17; found, 329.04.

**15**: (86%); Colorless oil; R*f* = 0.50 (PE/EA 5:2); ^1^H NMR (400 MHz, CDCl_3_) *δ*_H_(ppm): 9.94 (s, 1 H, CHO), 7.47 (d, *J* = 9.4 Hz, 2 H), 7.24–7.22 (d, *J* = 7.8 Hz, 1 H), 4.19 (m, 2 H), 3.89 (s, 3 H), 3.82–3.73 (m, 1 H), 3.53–3.41 (m, 1 H), 2.90–2.79 (quint, 1 H), 1.14–1.04 (d, *J* = 6.7 Hz, 3 H), 0.93 (t, *J* = 7.5 Hz, 3 H). ^13^C NMR (100 MHz, CDCl_3_) *δ*: 191.33, 172.39, 152.27, 145.26, 135.38, 125.02, 123.74, 111.00, 80.85, 77.43, 70.77, 56.29, 45.46, 40.98, 31.04, 30.24, 28.81, 13.66, 10.37. ESI-MS: calcd for C_19_H_26_O_6_ [M + Na]^+^, 373.16; found, 372.95.

**16**: (87%); Colorless oil; R*f* = 0.47 (PE/EA 5:3); ^1^H NMR (400 MHz, CDCl_3_) *δ*_H_(ppm): 7.58 (d, *J* = 8.1 Hz, 4 H), 7.44 (m, 4 H), 7.36 (m, 1 H), 5.23–5.14 (m, 2 H), 4.15 (m, 1 H), 4.05 (m, 1 H), 3.72 (m, 1 H), 2.66–2.58 (m, 1 H), 2.04–1.93 (m, 2 H), 1.65–1.58 (m, 4 H), 1.52–1.44 (m, 2 H), 1.16–1.15 (d, *J* = 7.0 Hz, 3 H), 0.92 (t, *J* = 7.4 Hz, 3 H). ^13^C NMR (100 MHz, CDCl_3_) *δ*: 174.86, 141.29, 140.96, 135.32, 129.00, 128.85, 127.61, 127.47, 127.34, 81.14, 70.65, 66.22, 45.64, 41.06, 30.92, 30.28, 28.94, 13.70, 10.33. ESI-MS: calcd for C_24_H_30_O_4_ [M + Na]^+^, 405.20; found, 407.20.

General procedure for compounds **17**–**19**: A solution of nonactic/homononactic acid (5.7 mg) and O-phenylenediamine (4.4 mg) in DCM/DMF (100 μL) was treated with HBTU (1 mg, 0.008 mmol), and the mixture was cooled at 0 °C in an ice bath. DIEA (10 μL) was added dropwise to the above solution. The reaction mixture was stirred at room temperature overnight. After the end of the reaction (monitoring by thin-layer chromatography), the resulting solution was quenched with water (2 mL) and extracted with ethyl acetate (2 mL×3). The combined organic layers were dried under vacuum and purified by column chromatography on silica gel.

**17**: (70%); Colorless oil; R*f* = 0.23 (DCM/MeOH 50:1); ^1^H NMR (400 MHz, CD_3_OD) *δ*_H_(ppm): 4.01–3.81 (m, 3 H), 3.70–3.58 (m, 2 H), 3.57–3.42 (m, 2 H), 2.96–2.86 (m, 1 H), 2.07–1.95 (m, 2 H), 1.75–1.46 (m, 10 H), 1.15 (d, *J* = 6.3 Hz, 3 H), 1.03 (d, *J* = 6.8 Hz, 3 H). ^13^C NMR (100 MHz, CD_3_OD) *δ*: 175.91, 82.76, 77.75, 66.16, 46.39, 44.35, 42.34, 32.25, 29.97, 27.86, 26.87, 25.62, 24.11, 14.67. ESI-MS: calcd for C_15_H_27_NO_3_ [M + Na]^+^, 292.19; found, 292.08.

**18**: (70%); Colorless oil; R*f* = 0.23 (DCM/MeOH 50:1); ^1^H NMR (400 MHz, CDCl_3_) *δ*_H_(ppm): 7.58 (d, *J* = 8.1 Hz, 4 H), 7.44 (m, 4 H), 7.36 (m, 1 H), 5.23–5.14 (m, 2 H), 4.15 (m, 1 H), 4.05 (m, 1 H), 3.72 (m, 1 H), 2.66–2.58 (m, 1 H), 2.04–1.93 (m, 2 H), 1.65–1.58 (m, 4 H), 1.52–1.44 (m, 2 H), 1.16–1.15 (d, *J* = 7.0 Hz, 3 H), 0.92 (t, *J* = 7.4 Hz, 3 H). ^13^C NMR (100 MHz, CDCl_3_) *δ*: 174.86, 141.29, 140.96, 135.32, 129.00, 128.85, 127.61, 127.47, 127.34, 81.14, 70.65, 66.22, 45.64, 41.06, 30.92, 30.28, 28.94, 13.70, 10.33. ESI-MS: calcd for C_24_H_30_O_4_ [M + Na]^+^, 405.20; found, 407.20.

**19**: (61%); White solid; R*f* = 0.50 (PE/EA 1:1); ^1^H NMR (400 MHz, CDCl_3_) *δ*_H_(ppm): 7.79 (m, 1 H), 7.54–7.43 (m, 1 H), 7.38–7.30 (m, 1 H), 7.18–6.96 (m, 1 H), 4.32–4.19 (m, 1 H), 3.99–3.90 (q, *J* = 7.1 Hz, 1 H), 3.76–3.61 (m, 1 H), 2.75–2.68 (quint, 1 H), 1.13–1.08 (d, *J* = 6.6 Hz, 3 H), 1.01 (t, *J* = 7.4 Hz, 3 H). ^13^C NMR (100 MHz, CDCl_3_) *δ*:173.65, 126.91, 124.55, 121.74, 120.25, 80.58, 77.43, 69.74, 46.57, 40.75, 31.65, 31.01, 30.36, 14.58, 10.43. ESI-MS: calcd for C_18_H_24_N_2_O_3_S [M + Na]^+^, 371.14; found, 371.01.

Silylether **20**: Imidazole (6 mg, 0.088 mmol) and t-Butyldimethylchlorosilane (8.4 mg, 0.056 mmol) were added to a solution of homononactic acid (3.4 mg, 0.016 mmol) in DMF (50 μL). After stirring for 16h at room temperature, the reaction mixture was quenched with water (2 mL) and extracted with ethyl acetate (2 mL×3). The combined organic layers were dried in vacuo and purified by column chromatography on silica gel (DCM/MeOH 20:1) gave **20** (5 mg, 96%) as a colorless oil. R*f* = 0.75 (DCM/MeOH 10:1). ^1^H NMR (400 MHz, CDCl_3_) *δ*_H_(ppm): 4.04 (m, 1 H), 3.92 (q, *J* = 7.48 Hz, 1 H), 3.76–3.81 (m, 1 H), 2.55–2.47 (quint, 1 H), 2.08–1.98 (m, 2 H), 1.67–1.54 (m, 4 H), 1.52–1.44 (m, 2 H), 1.18 (d, *J* = 7.08 Hz, 3 H), 0.88 (s, 9 H), 0.84 (d, *J* = 7.5 Hz, 3 H), 0.04 (s, 6 H). ^13^C NMR (100 MHz, CDCl_3_) *δ* 177.68, 70.75, 45.09, 42.79, 31.51, 30.87, 29.65, 26.14, 18.34, 13.63, 9.11, −4.24, −4.58. ESI-MS: calcd for C_17_H_34_O_4_Si [M + H]^+^, 330.54; found, 331.26.

Benzimidazole derivative **21**: Compound **18** (4.8 mg) was dissolved in 200 μL acetate, and the mixture was reflux for 8 h. After the end of the reaction (monitoring by thin-layer chromatography), the resulting solution was quenched with saturated sodium bicarbonate (2 mL) and extracted with ethyl acetate (2 mL×3). The combined organic layers were dried in vacuo and purified by column chromatography on silica gel (PE/EA 1:2–1:3), which gave 21 (3.7 mg, 82%) as a white solid. R*f* = 0.13 (PE/EA 1:2). ^1^H NMR (400 MHz, CDCl_3_) *δ*_H_(ppm): 7.76–7.65 (m, 2 H), 7.34–7.28 (m, 2 H), 4.25–4.14 (m, 1 H), 4.09 (m, 1 H), 3.87–3.77 (m, 1 H), 3.44 (m, 1 H), 1.51 (d, *J* = 6.7 Hz, 3 H), 0.96 (t, *J* = 7.4 Hz, 3 H). ^13^C NMR (100 MHz, CDCl_3_) *δ*: 155.51, 133.88, 124.44, 114.63, 81.49, 77.43, 70.83, 40.77, 38.66, 31.64, 30.45, 30.41, 30.23, 30.21, 29.91, 17.03, 16.99, 10.45. ESI-MS: calcd for C_18_H_24_N_2_O_3_S [M + Na]^+^, 371.14; found, 371.01.

### 3.7. Cytotoxicity Assays (MTT Assays)

The cytotoxic activity of the compounds on cancer cell lines, including HT-29, MCF-7, A375, K562, andNCM460cell lines, was measured using the MTT assay with a small modification. Briefly, cells were plated in 96-well plates at a density of 1 × 10^4^ cells per well and allowed to grow overnight, then the medium was replaced with fresh RPMI1640 medium containing serially diluted compounds, and the cells were further incubated at 37 °C with 5% CO_2_ for 48 h. In this experiment, **1** and **6** were diluted into six different series concentrations from 10 μg/mL to 0.0097 μg/mL with 4× dilution each, while **2** and **3** were diluted from 500 μg/mL to 2.06 μg/mL, and **4**, **5**, and **7** from 300 μg/mL to 1.23 μg/mL with 3× dilution each. Paclitaxel, a clinical medication used as a positive control, was diluted from 100 μg/mL to 3.125 μg/mL with two series dilutions. HT-29, MCF-7, and A375 were all cultured in serum-free RPMI1640, while the K562 was cultured in DMEM containing FBS throughout this experiment. The culture medium was removed after the incubation, and then 100 μL of the 5 mg/mL MTT solution was added to each well and further incubated at 37 °C for 4 h. Finally, the supernatants were removed, and 150 μL of dimethyl sulfoxide (DMSO) was added to each well. After shaking the plates for 10 min at room temperature, the optical density value was measured spectrophotometrically at 570 nm using the microplate reader. The IC_50_ value (50% inhibitory concentration) was determined to evaluate the cell viability of these cell lines. In this experiment, Graph Pad Prism 5software (San Diego, CA, USA) was used to calculate the half inhibitory concentration (IC_50_) values. Each experiment was independently repeated at least three times and represented as the means ± standard deviation (SD).

## 4. Conclusions

In summary, a new compound (**1**) and six known compounds, including nonactic acid (**2**), homononactic acid (**3**), ethyl homononactate (**4**), homononactylhomononactate (**5**), valinomycin (**6**), and cyclo-(Pro-Leu) (**7**), were isolated from the culture broth of *Streptomyces* sp. BM-8 residing in the feces of *Equus quagga*. Subsequently, 13 derivatives of nonactic/homononactic acid were synthesized. In this study, **6** showed significant cytotoxic activities against all tested cell lines, while **1** exhibited less activity toward these cells. Some synthesized compounds showed higher toxicity towards the nontumorigenic line and the absence of a structure–activity relationship, indicating that these compounds may have broad cellular toxicity toward the cell lines tested. The synthesized compounds did not show attractive selectivity in the cytotoxic assay, suggesting that more synthesis strategies should be proposed. Since the dimeric nonactic acid show less antibacterial and antifungal activity than that of dimeric dinactin [14], it might be worth synthesizing such kinds of cyclic analogues with nonactic acid or homononactic acid in further research. Nevertheless, these results suggested that microbes inhabiting the animal feces may represent an important source for new and bioactive natural compounds.

## Figures and Tables

**Table 1:** NMR spectral data for **1** in CD_3_OD.

C/H	*δ*_H_ (Mult, *J* in Hz) ^a^	*δ*_C_^b^	HMBC	COSY
1	-	184.5, C ^c^		
2	2.29 (quint, *J* = 6.16, 1 H)	48.3, CH		H-11, H-3
3	3.49–3.54 (m, 1 H)	74.6, CH		H-2, H-4b
4a	1.79–1.86 (m, 1 H)	30.3, CH_2_		H-3, H-5
4b	1.62–1.71 (m, 1 H)			
5	2.62–2.65 (m, 2 H)	41.0, CH_2_	C-6, C-4	H-4a, H-4b
6	-	212.9, C		
7	2.56 (d, *J* = 6.36, 2 H)	50.9, CH_2_	C-6, C-8, C-9	H-8
8	3.92–3.98 (m, 1 H)	70.4, CH		H-9, H-7
9	1.41–1.51 (m, 2 H)	31.2, CH_2_	C-10, C-8, C-7	H-10, H-8
10	0.94 (t, *J* = 7.40, 3 H)	10.3, CH_3_	C-9, C-8	H-9
11	1.18 (d, *J* = 7.04, 3 H)	15.9, CH_3_	C-1, C-2, C-3	H-2

^a^ 400 MHz. ^b^ 100 MHz.^c^ obtained from the HMBC spectrum.

**Table 2:** ^1^H NMR spectral data for **2**–**5** in CDCl_3_.

Position	2	3	4	5
	*δ*_H_ (mult, *J* in Hz) ^a^	*δ*_H_ (mult, *J* in Hz)	*δ*_H_ (mult, *J* in Hz)	*δ*_H_ (mult, *J* in Hz)
2	2.49 (quint, *J* = 7.04, 1 H)	2.51 (quint, *J* = 7.08, 1 H)	2.52 (quint, *J* = 7.04, 1 H)	2.49 (quint, *J* = 7.0, 1 H)
3	3.96–4.01 (m, 1 H)	3.96–4.02 (m, 1 H)	3.96–4.01 (m, 1 H)	3.91–4.02 (m, 1 H)
4	1.69–1.73 (m, 2 H)	1.44–1.73 (m, 1 H); 1.64–1.69 (m, 1 H)	1.94–2.04 (m, 1 H); 1.60–1.68 (m, 1 H)	1.97–2.02 (m, 1 H); 1.66–1.70 (m, 1 H)
5	1.96–2.08 (m, 2 H)	1.97–2.09 (m, 1 H); 1.64–1.69 (m, 1 H)	1.94–2.04 (m, 1 H); 1.60–1.68 (m, 1 H)	2.04–2.06 (m, 1 H); 1.57–1.59 (m, 1 H)
6	4.16–4.23 (m, 1 H)	4.19–4.25 (m, 1 H)	4.12–4.19 (m, 1 H)	3.91–4.02 (m, 1 H)
7	1.69–1.73 (m, 2 H)	1.44–1.73 (m, 2 H)	1.68–1.77 (m, 2 H)	1.75–1.78 (m, 2 H)
8	4.05–4.09 (m, 1 H)	3.75–3.81 (m, 1 H)	3.72–3.75 (m, 1 H)	4.98 (quint, *J* = 6.12, 1 H)
9	1.22 (d, *J* = 6.32, 3 H)	1.44–1.73 (m, 2 H)	1.43–1.54 (m, 2 H)	1.55–1.61 (m, 2 H)
10	1.16 (d, *J* = 7.00, 3 H)	0.93 (t, *J* = 7.44, 3 H)	0.93 (t, *J* = 7.44, 3 H)	0.88 (t, *J* = 7.44, 3 H)
11		1.17 (t, *J* = 7.00, 3 H)	1.12 (d, *J* = 7.44, 3 H)	1.17 (d, *J* = 7.04, 3 H)
1′			4.12–4.19 (m, 2 H)	-
2′			1.26 (t, *J* = 7.12, 3 H)	2.49 (quint, *J* = 7.0, 1 H)
3′				4.91–3.98 (m, 1 H)
4′				1.97–2.02 (m, 1 H); 1.59–1.61 (m, 1 H)
5′				2.04–2.06 (m, 1 H); 1.61–1.64 (m, 1 H)
6′				4.14–4.17 (m, 1 H)
7′				1.67–1.71 (m, 2 H)
8′				3.71–3.77 (m, 1 H)
9′				1.43–1.55 (m, 2 H)
10′				0.94 (t, *J* = 6.60, 3 H)
11′				1.13 (d, *J* = 7.04, 3 H)

^a^ 400 MHz, *δ* in ppm.

**Table 3:** ^13^C NMR spectral data for **2**–**5** in CDCl_3_.

Position	2	3	4	5
	*δ*_C_^a^	*δ*_C_	*δ*_C_	*δ*_C_
1	178.1, C	177.8, C	175.1, C	176.6, C
2	45.5, CH	45.5, CH	45.5, CH	45.2, CH
3	81.3, CH	81.2, CH	81.3, CH	80.9, CH
4	29.2, CH_2_	29.3, CH_2_	29.0, CH_2_	29.6, CH_2_
5	30.7, CH_2_	30.8, CH_2_	30.8, CH_2_	31.2, CH_2_
6	77.3, CH	77.5, CH	77.5, CH	76.9, CH
7	43.0, CH_2_	40.9, CH_2_	40.8, CH_2_	40.4, CH_2_
8	65.3, CH	70.6, CH	70.7, CH	73.2, CH
9	23.2, CH_3_	30.1, CH_2_	30.2, CH_2_	27.5, CH_2_
10	13.9, CH_3_	10.3, CH_3_	10.4, CH_3_	9.6, CH_3_
11		13.9, CH_3_	13.7, CH_3_	13.8, CH_3_
1′			60.7, CH_2_	174.9, C
2′			14.4, CH_3_	45.7, CH
3′				81.3, CH
4′				29.0, CH_2_
5′				30.6, CH_2_
6′				77.3, CH
7′				40.7, CH_2_
8′				70.6, CH
9′				30.1, CH_2_
10′				10.3, CH_3_
11′				14.2, CH_3_

^a^ 100 MHz, *δ* in ppm.

**Table 4:** Cytotoxic activity of **1**–**21** against various cell lines.

Compound	IC_50_ (Means ± SD, μM) ^a^				
	HT-29 ^b^	MCF-7 ^c^	A375 ^d^	K562 ^e^	NCM460 ^f^
**1**	1.98 ± 1.04	5.44 ± 1.33	44.95 ± 1.21	1.56 ± 0.21	27.93 ± 4.70
**2**	44.35 ± 2.77	289.34 ± 3.54	185.87 ± 1.27	83.64 ± 2.54	1604.55 ± 14.41
**3**	39.94 ± 3.22	99.10 ± 2.66	146.1 ± 2.01	57.04 ± 1.88	1027.36 ± 8.01
**4**	11.79 ± 1.83	39.58 ± 2.57	65.09 ± 0.62	12.32 ± 0.54	249.26 ± 4.22
**5**	38.09 ± 2.35	16.69 ± 1.08	30.66 ± 0.93	14.15 ± 0.47	33.31 ± 3.31
**6**	1.22 ± 0.24	0.06 ± 0.01	1.85 ± 0.22	0.04 ± 0.01	1.66 ± 0.19
**7**	101.56 ± 2.66	78.78 ± 1.95	51.13 ± 4.73	21.72 ± 0.35	775.86 ± 11.43
**8**	441.30 ± 1.65	847.39 ± 39.52	445.93 ± 4.10	NA ^h^	164.26 ± 3.09
**9**	113.45 ± 3.68	171.09 ± 5.08	115.43 ± 3.66	69.38 ± 3.90	28.99 ± 3.68
**10**	326.76 ± 1.40	416.54 ± 3.90	314.60 ± 0.57	285.13 ± 2.44	109.23 ± 3.60
**11**	43.16 ± 2.84	34.06 ± 1.87	24.29 ± 0.03	38.25 ± 0.70	44.21 ± 0.64
**12**	209.05 ± 3.25	485.58 ± 3.18	127.43 ± 6.65	186.91 ± 4.57	150.15 ± 4.03
**13**	321.67 ± 4.17	362.39 ± 0.96	217.56 ± 0.99	312.53 ± 3.29	299.32 ± 1.77
**14**	73.24 ± 1.05	111.18 ± 3.89	89.92 ± 1.09	41.38 ± 2.84	21.05 ± 3.66
**15**	531.44 ± 5.46	NT ^i^	174.84 ± 6.11	175.13 ± 3.28	NT
**16**	165.08 ± 1.23	155.92 ± 3.56	67.32 ± 0.25	105.36 ± 2.58	109.90 ± 1.36
**17**	562.52 ± 7.35	389.63 ± 10.00	199.11 ± 1.90	338.96 ± 1.23	179.78 ± 7.88
**18**	235.65 ± 1.31	302.35 ± 1.83	107.99 ± 5.22	402.94 ± 0.69	55.72 ± 7.65
**19**	169.37 ± 4.20	270.26 ± 1.09	128.5 ± 0.48	NA	80.06 ± 3.82
**20**	356.36 ± 7.67	284.27 ± 1.24	215.00 ± 0.76	323.33 ± 5.94	37.00 ± 1.39
**21**	205.28 ± 0.63	355.90 ± 1.1	369.5 ± 3.39	301.82 ± 1.32	22.50 ± 3.09
Paclitaxel ^g^	5.69 ± 1.32 nM	13.78 ± 2.12 nM	45.80 ± 1.79 nM	9.31 ± 1.88 nM	18.25 ± 2.34 nM

^a^ IC_50_ means 50% inhibitory concentration, and expressed as the means ± standard deviation (SD), each of which was repeated at least three times independently; ^b^ HT-29: human colon cancer cell line; ^c^ MCF-7: human breast cancer cell line; ^d^ A375: human malignant melanoma cell line; ^e^ K562: human chronic myeloid leukemia cell line, and it was cultured in RPMI1640 containing FBS while the other cell lines were all cultured in medium without the addition of FBS; ^f^ NCM460: human normal colon epithelial cell line; ^g^ Paclitaxel was used as a positive control; ^h^ NA: not active; ^i^ NT: not tested.

## Supplementary Materials

The following are available online.

## Sample Availability

Samples of the compounds are available from the authors.
