# Supplementary material for: Secondary Metabolites with Cytotoxic Activities from Streptomyces sp. BM-8 Isolated from the Feces of Equus quagga
Source: Molecules. 2021 Dec 13;26(24):7556. doi: 10.3390/molecules26247556 (PMC8704880; doi:10.3390/molecules26247556)
Supplement: Supplementary file 1 [file molecules-26-07556-s001.zip › molecules-1462041-supplementary.pdf]

Supporting Information

Secondary Metabolites with Cytotoxic Activities from *Streptomyces* sp. BM-8 Isolated from the Feces of *Equus quagga*

Shengsheng Lu[1,2#], Jianan Hu[1#], Xi Xie[3#], Runhong Zhou[1], Fangfang L[1], Ruifeng Huang[1], Jian He[1,*]

[1]*Group of Peptides and Natural Products Research, School of Pharmaceutical Sciences, Southern Medical University, 1838 Guangzhou Avenue North, Guangzhou 510515, People's Republic of China*

[2]*Department of Pharmacy, Affiliated Tumor Hospital of Guangxi Medical University, Nanning 530021, Guangxi, People's Republic of China*

[3]*Guangdong Provincial Key Laboratory of Emergency Test for Dangerous Chemicals, China National Analytical Center 510070, People's Republic of China*

[#] These authors are contributed equally.

*To whom the correspondence should be addressed.

Jian He Tel: +86-20-61648717, E-mail: jianhe@smu.edu.cn

© 2019 ACG Publications. All rights reserved.

Table of Contents	Page
**Figure S1.** ^{1}H NMR spectrum of **1** in CD_3OD	4
**Figure S2.** ^{13}C NMR and DEPT spectrum of **1** in CD_3OD	4
**Figure S3.** ^{1}H-^{1}H COSY spectrum of **1** in CD_3OD	5
**Figure S4.** HMQC spectrum of **1** in CD_3OD	5
**Figure S5.** HMBC spectrum of **1** in CD_3OD	6
**Figure S6.** **(+)**-HRESI-MS spectrum of **1**	6
**Figure S7.** **(-)**-HRESI-MS spectrum of **1**	7
**Figure S8.** ^{1}H NMR spectrum of **2** in $CDCl_3$	8
**Figure S9.** ^{13}C NMR and DEPT spectrum of **2** in $CDCl_3$	8
**Figure S10.** **(+)**-ESI-MS spectrum of **2**	9
**Figure S11.** ^{1}H NMR spectrum of **3** in $CDCl_3$	9
**Figure S12.** ^{13}C NMR and DEPT spectrum of **3** in $CDCl_3$	10
**Figure S13.** **(-)**-ESI-MS spectrum of **3**	10
**Figure S14.** ^{1}H NMR spectrum of **4** in $CDCl_3$	11
**Figure S15.** ^{13}C NMR and DEPT spectrum of **4** in $CDCl_3$	11
**Figure S16.** **(+)**-ESI-MS spectrum of **4**	12
**Figure S17.** ^{1}H NMR spectrum of **5** in $CDCl_3$	12
**Figure S18.** ^{13}C NMR and DEPT spectrum of **5** in $CDCl_3$	13
**Figure S19.** **(+)**-ESI-MS spectrum of **5**	13
**Figure S20.** ^{1}H NMR spectrum of **6** in $CDCl_3$	14
**Figure S21.** ^{13}C NMR and DEPT spectrum of **6** in $CDCl_3$	14
**Figure S22.** **(+)**-ESI-MS spectrum of **6**	15
**Figure S23.** ^{1}H NMR spectrum of **7** in CD_3OD	15
**Figure S24.** ^{13}C NMR and DEPT spectrum of **7** in CD_3OD	16
**Figure S25.** **(+)**-ESI-MS spectrum of **7**	16
**Figure S26.** ^{1}H NMR spectrum of **8** in CD_3Cl_3	17

© 2019 ACG Publications. All rights reserved.

Figure S27. ^{13}C NMR spectrum of **8** in CD_3Cl_3 17

Figure S28. ^{1}H NMR spectrum of **9** in CD_3Cl_3 18

Figure S29. ^{13}C NMR spectrum of **9** in CD_3Cl_3 18

Figure S30. ^{1}H NMR spectrum of **10** in CD_3Cl_3 19

Figure S31. ^{13}C NMR spectrum of **10** in CD_3Cl_3 19

Figure S32. ^{1}H NMR spectrum of **11** in CD_3Cl_3 20

Figure S33. ^{13}C NMR spectrum of **11** in CD_3Cl_3 20

Figure S34. ^{1}H NMR spectrum of **12** in CD_3Cl_3 21

Figure S35. ^{13}C NMR spectrum of **12** in CD_3Cl_3 21

Figure S36. ^{1}H NMR spectrum of **13** in CD_3Cl_3 22

Figure S37. ^{13}C NMR spectrum of **13** in CD_3Cl_3 22

Figure S38. ^{1}H NMR spectrum of **14** in CD_3Cl_3 23

Figure S39. ^{13}C NMR spectrum of **14** in CD_3Cl_3 23

Figure S40. ^{1}H NMR spectrum of **15** in CD_3Cl_3 24

Figure S41. ^{13}C NMR spectrum of **15** in CD_3Cl_3 24

Figure S42. ^{1}H NMR spectrum of **16** in CD_3Cl_3 25

Figure S43. ^{13}C NMR spectrum of **16** in CD_3Cl_3 25

Figure S44. ^{1}H NMR spectrum of **17** in CD_3Cl_3 26

Figure S45. ^{13}C NMR spectrum of **17** in CD_3Cl_3 26

Figure S46. ^{1}H NMR spectrum of **19** in CD_3Cl_3 27

Figure S47. ^{13}C NMR spectrum of **19** in CD_3Cl_3 27

Figure S48. ^{1}H NMR spectrum of **20** in CD_3Cl_3 28

Figure S49. ^{13}C NMR spectrum of **20** in CD_3Cl_3 28

Figure S50. ^{1}H NMR spectrum of **21** in CD_3Cl_3 29

Figure S51. ^{13}C NMR spectrum of **21** in CD_3Cl_3 29

© 2019 ACG Publications. All rights reserved.

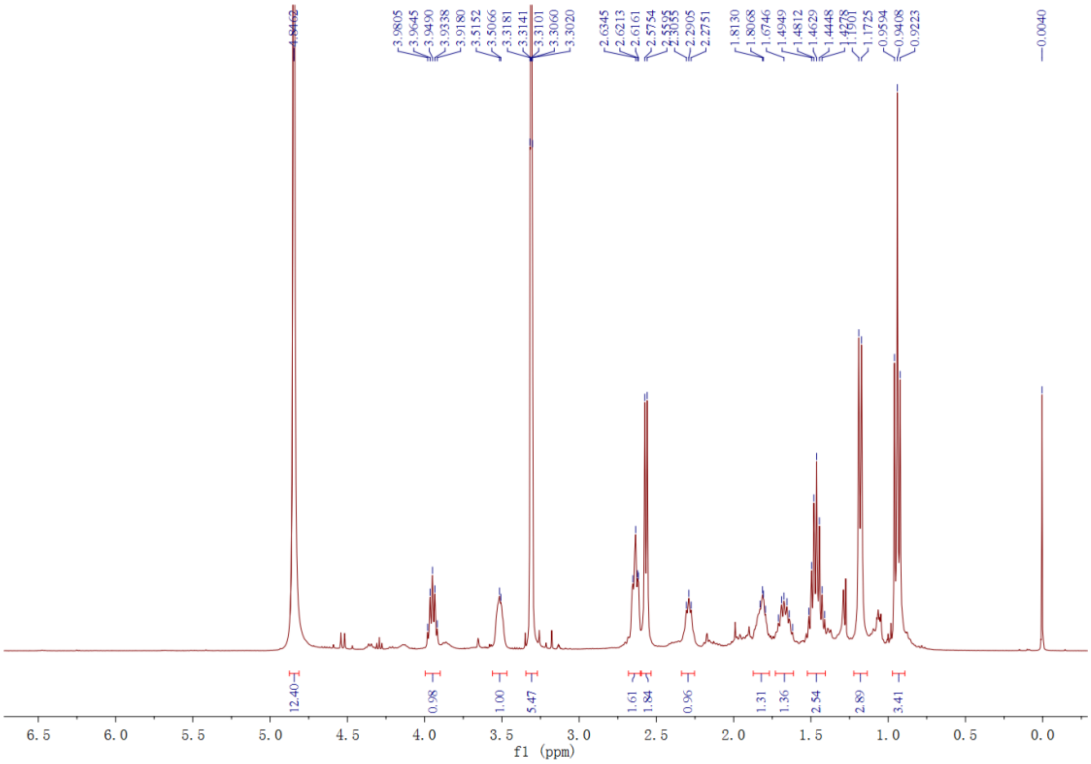

Figure S1. ^{1}H NMR spectrum of **1** in CD_3OD

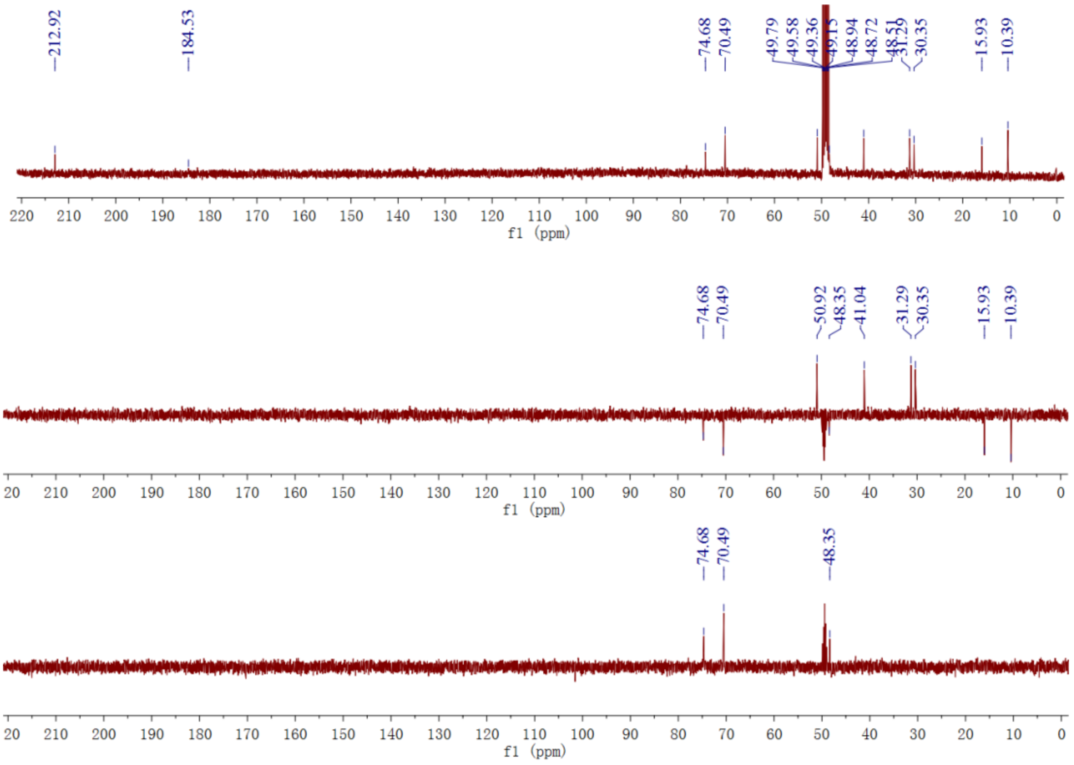

Figure S2. ^{13}C NMR and DEPT spectrum of **1** in CD_3OD

© 2019 ACG Publications. All rights reserved.

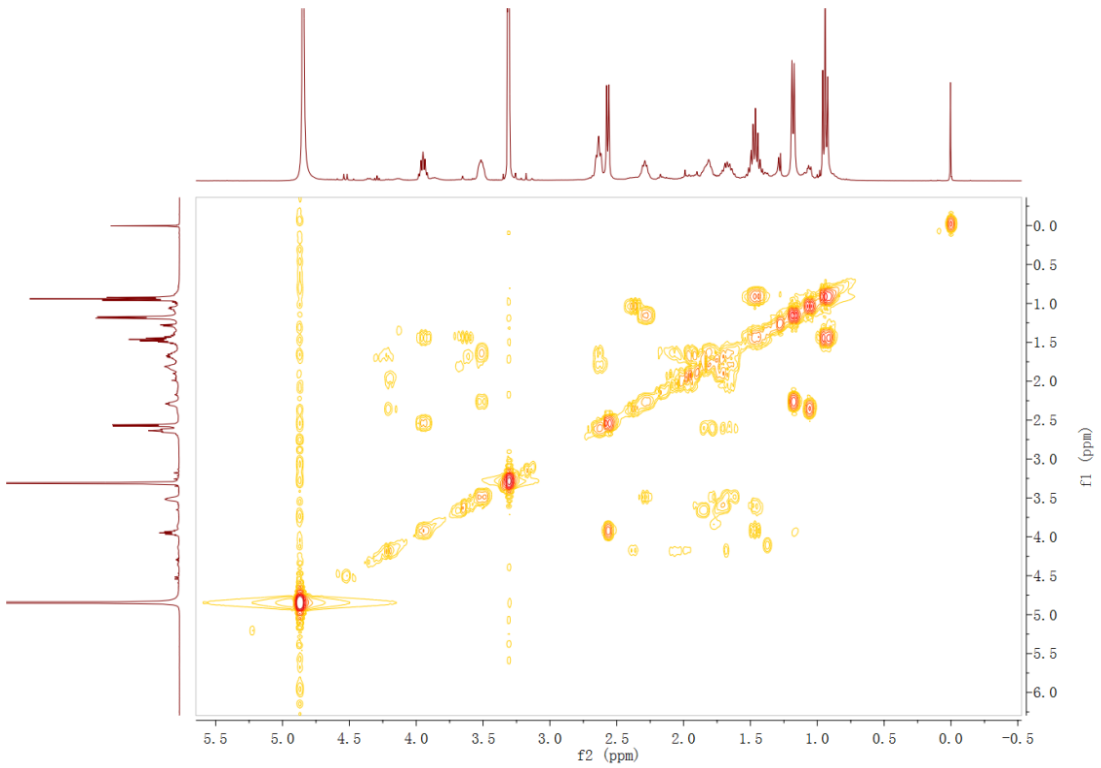

Figure S3. ^1H-^1H COSY spectrum of **1** in CD_3OD

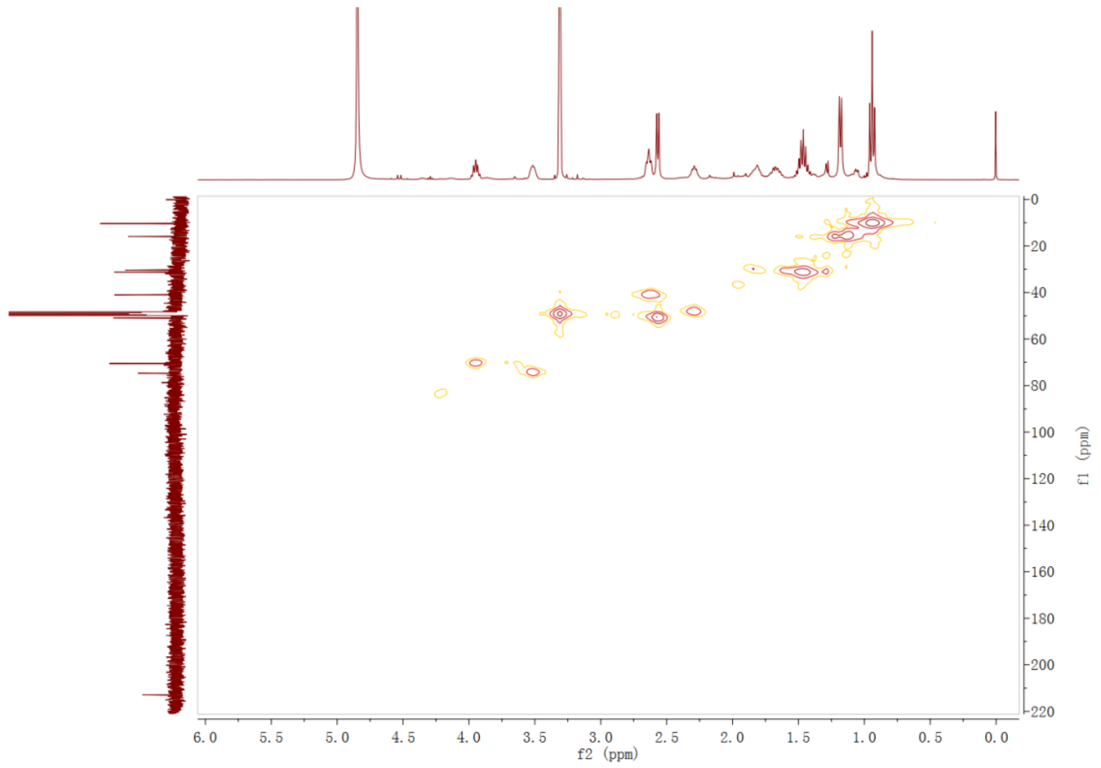

Figure S4. HMQC spectrum of **1** in CD_3OD

© 2019 ACG Publications. All rights reserved.

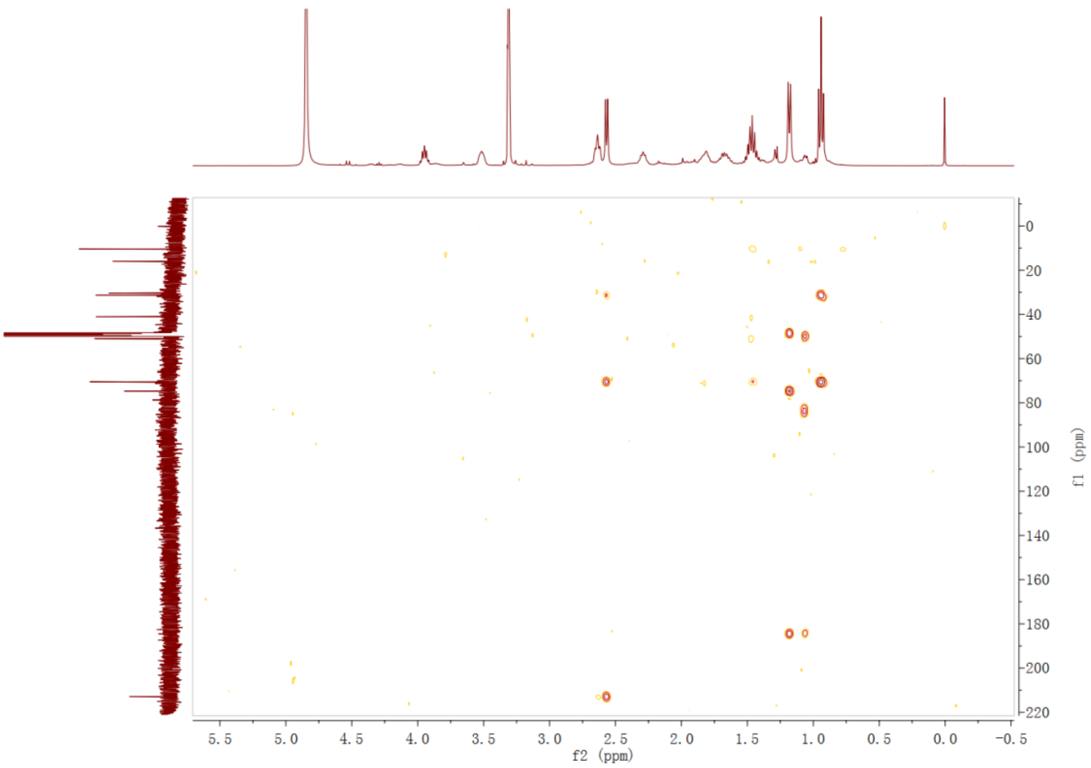

Figure S5. HMBC spectrum of **1** in CD_3OD

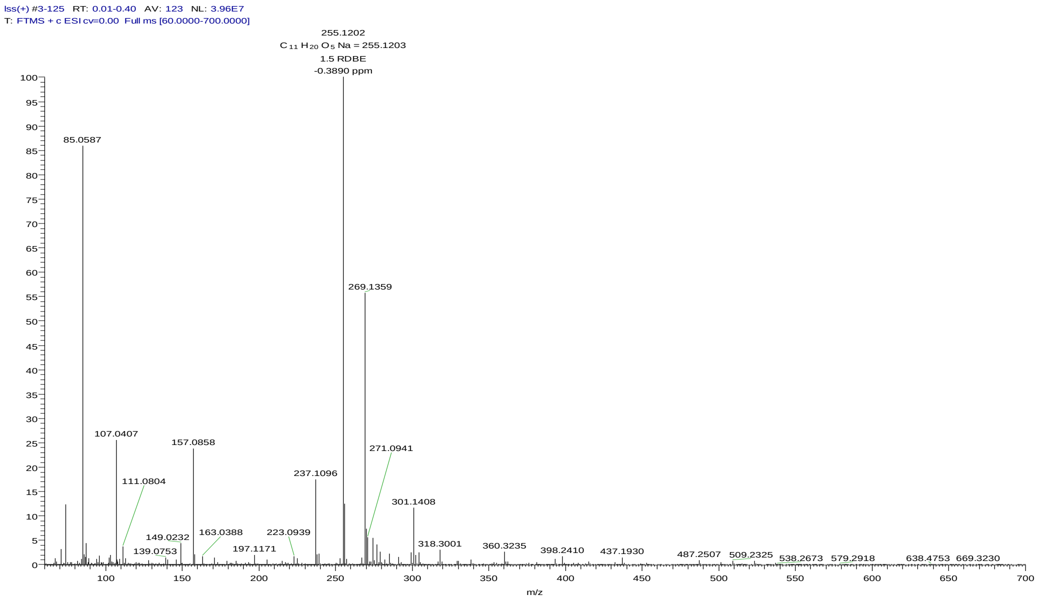

Figure S6. (+)-HRESI-MS spectrum of **1**

© 2019 ACG Publications. All rights reserved.

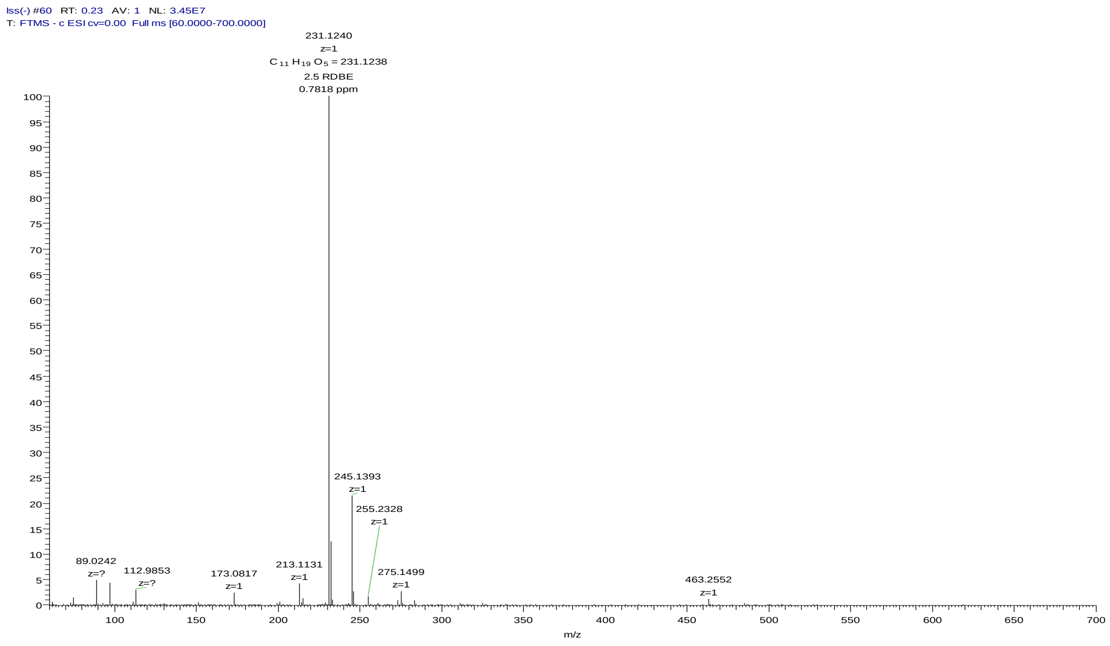

Figure S7. **(-)**-HRESI-MS spectrum of **1**

© 2019 ACG Publications. All rights reserved.

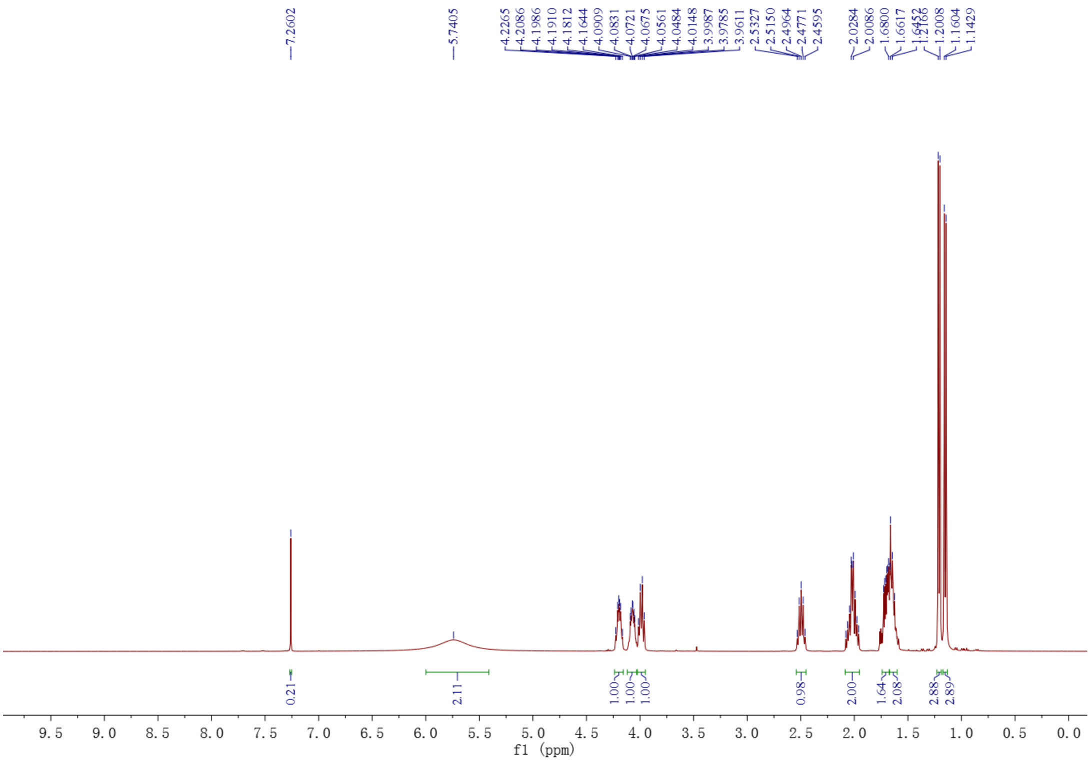

Figure S8. ^{1}H NMR spectrum of **2** in $CDCl_3$

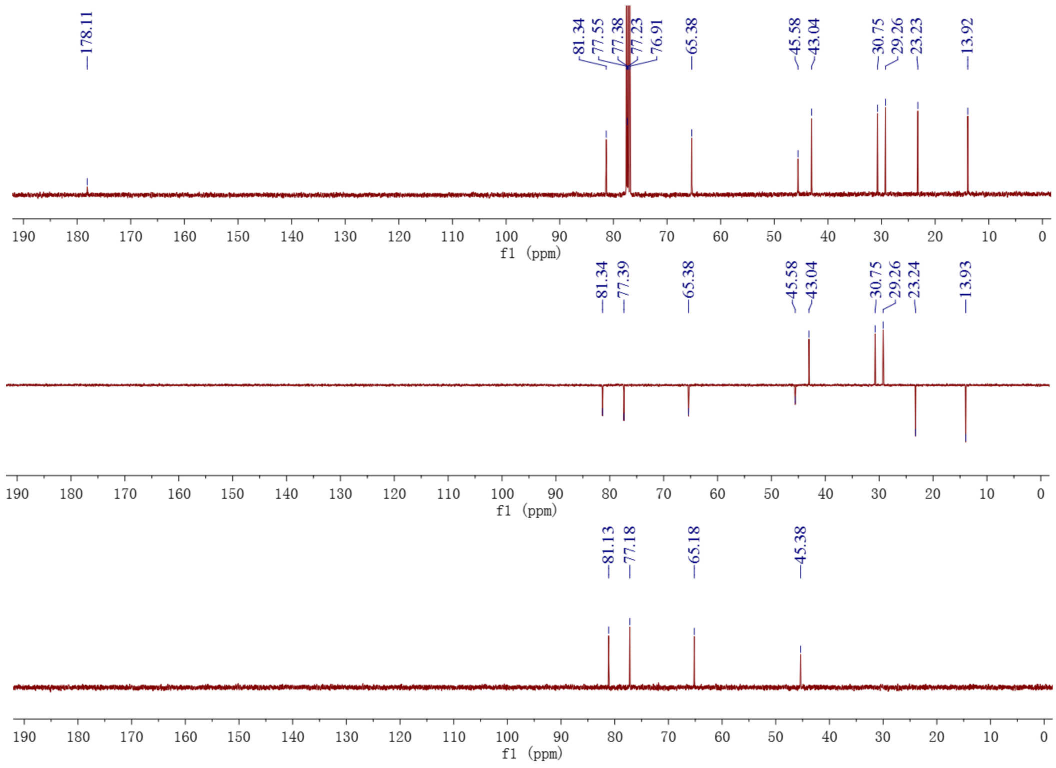

Figure S9. ^{13}C NMR and DEPT spectrum of **2** in $CDCl_3$

© 2019 ACG Publications. All rights reserved.

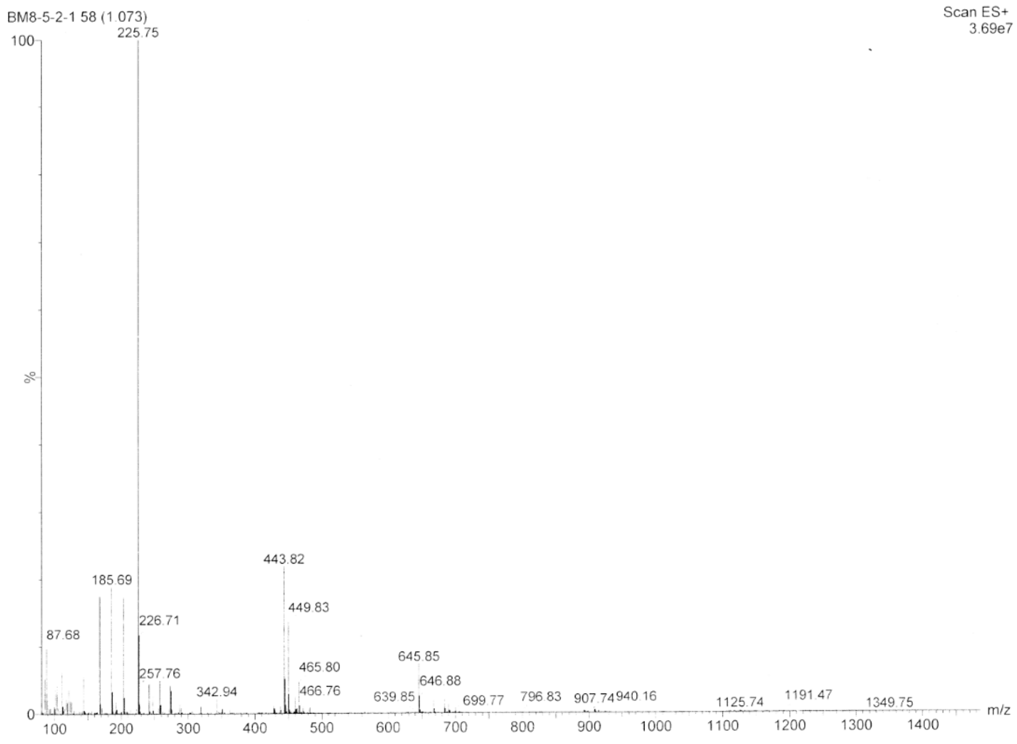

Figure S10. **(+)**-ESI-MS spectrum of **2**

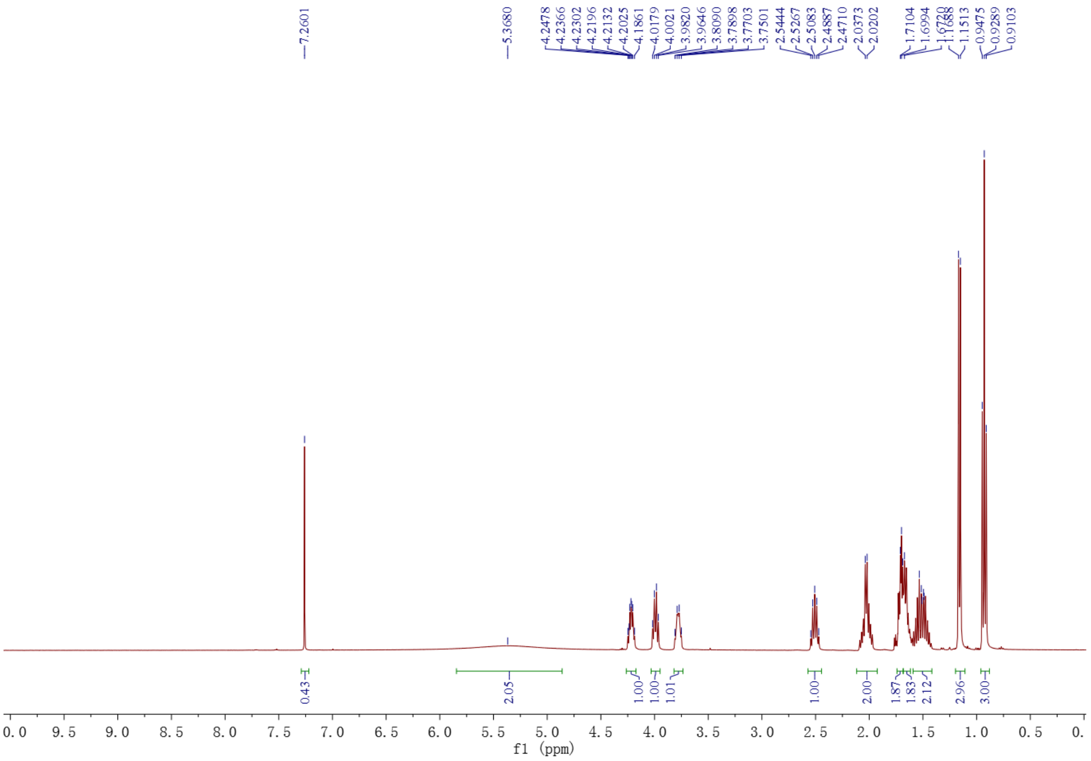

Figure S11. ^{1}H NMR spectrum of **3** in $CDCl_3$

© 2019 ACG Publications. All rights reserved.

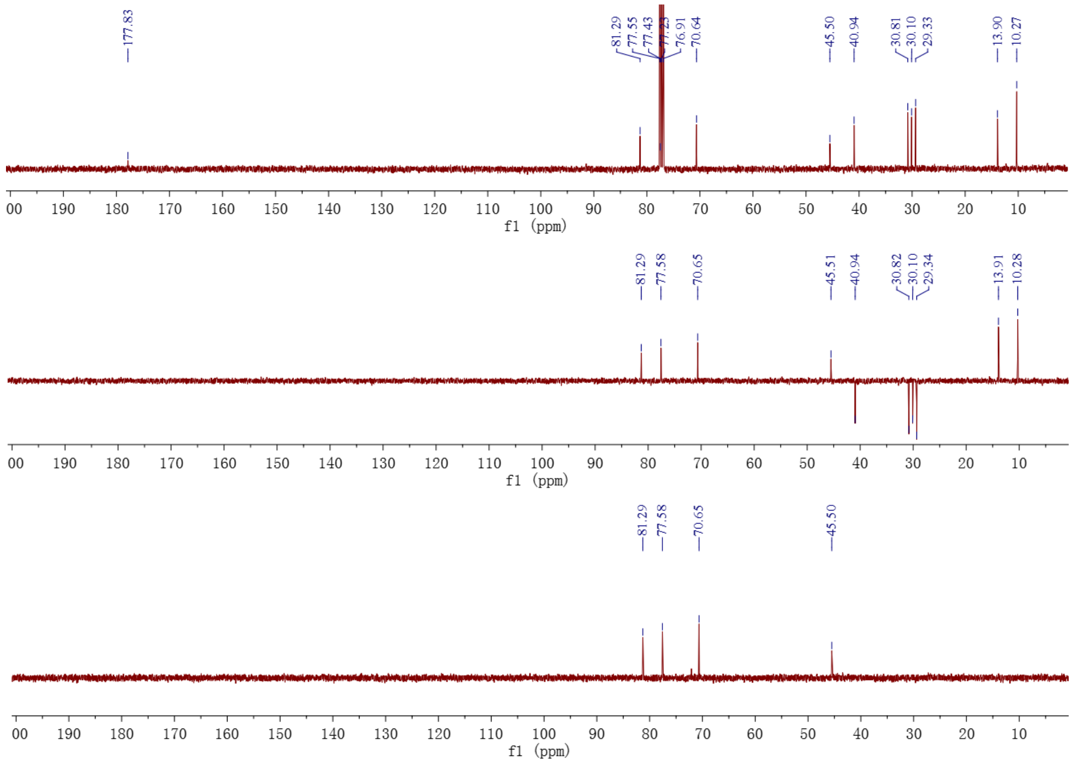

Figure S12. ^{13}C NMR and DEPT spectrum of **3** in $CDCl_3$

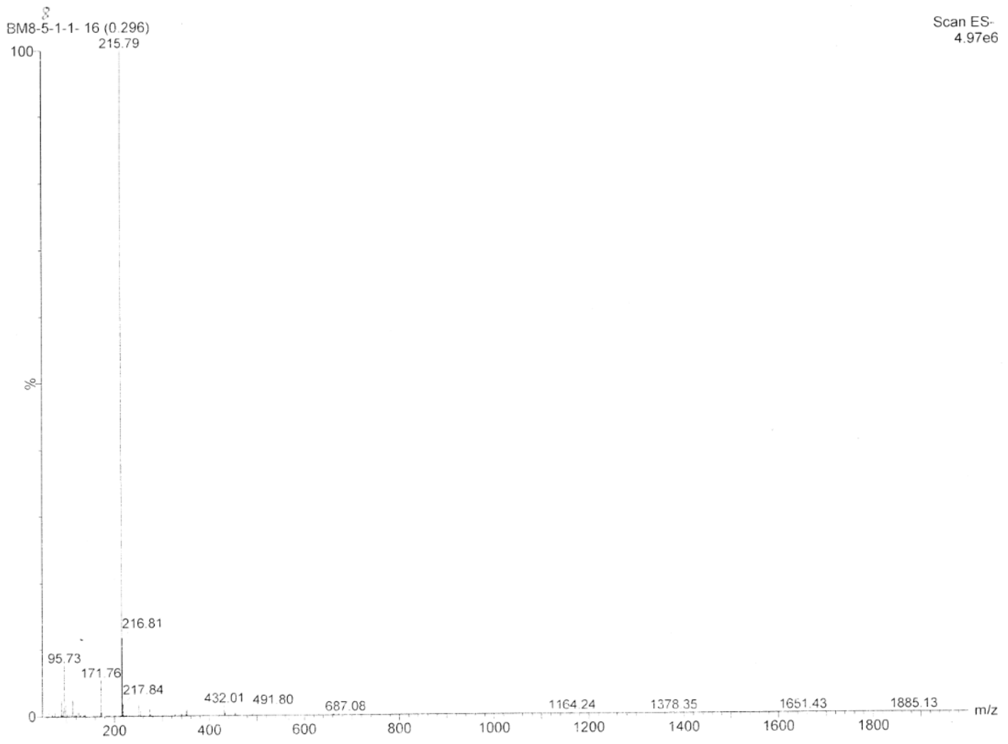

Figure S13. (-)-ESI-MS spectrum of **3**

© 2019 ACG Publications. All rights reserved.

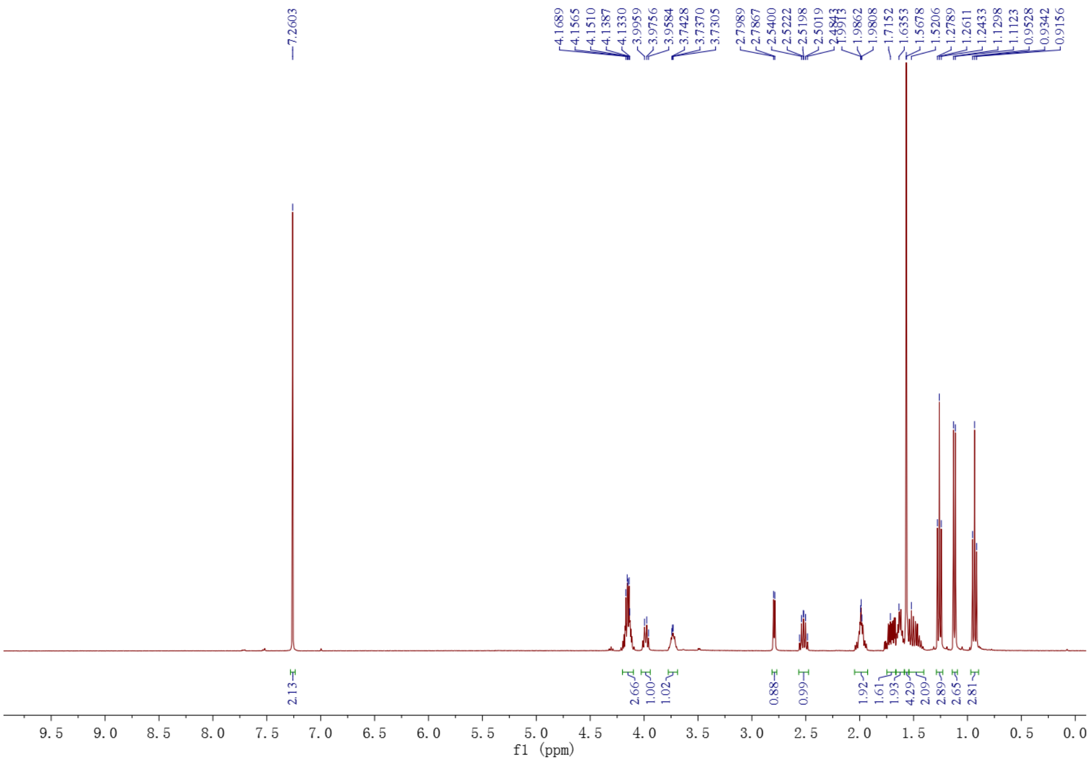

Figure S14. ^{1}H NMR spectrum of **4** in $CDCl_3$

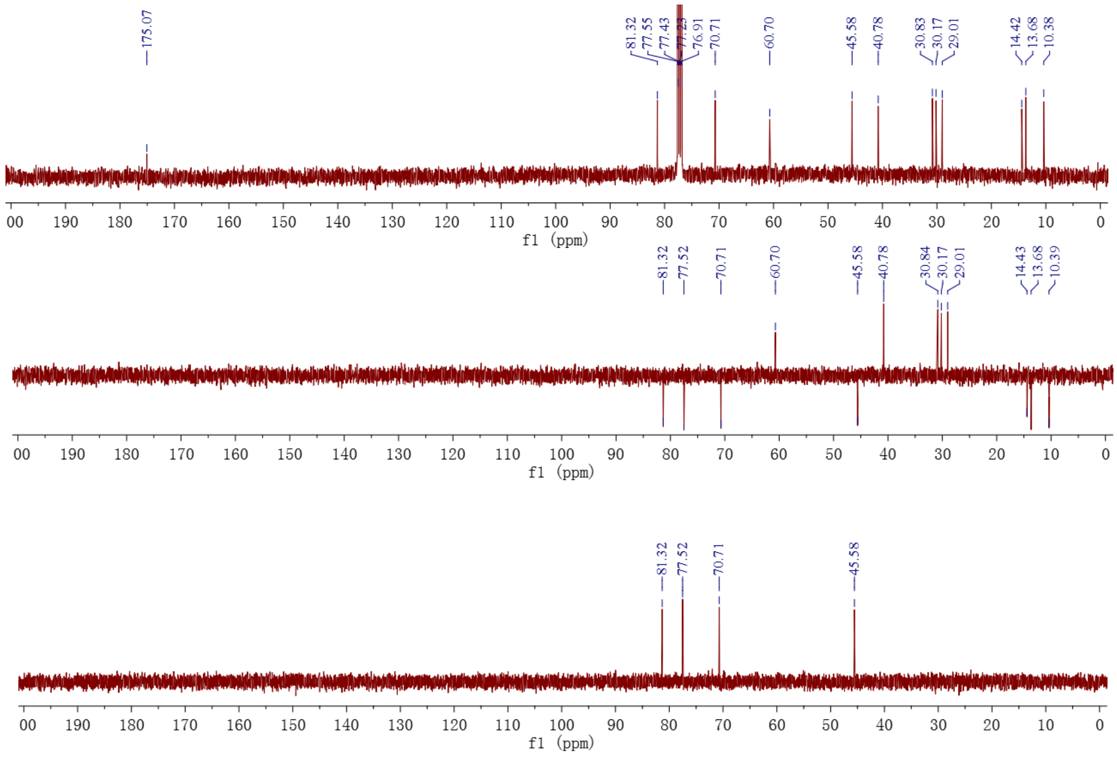

Figure S15. ^{13}C NMR and DEPT spectrum of **4** in $CDCl_3$

© 2019 ACG Publications. All rights reserved.

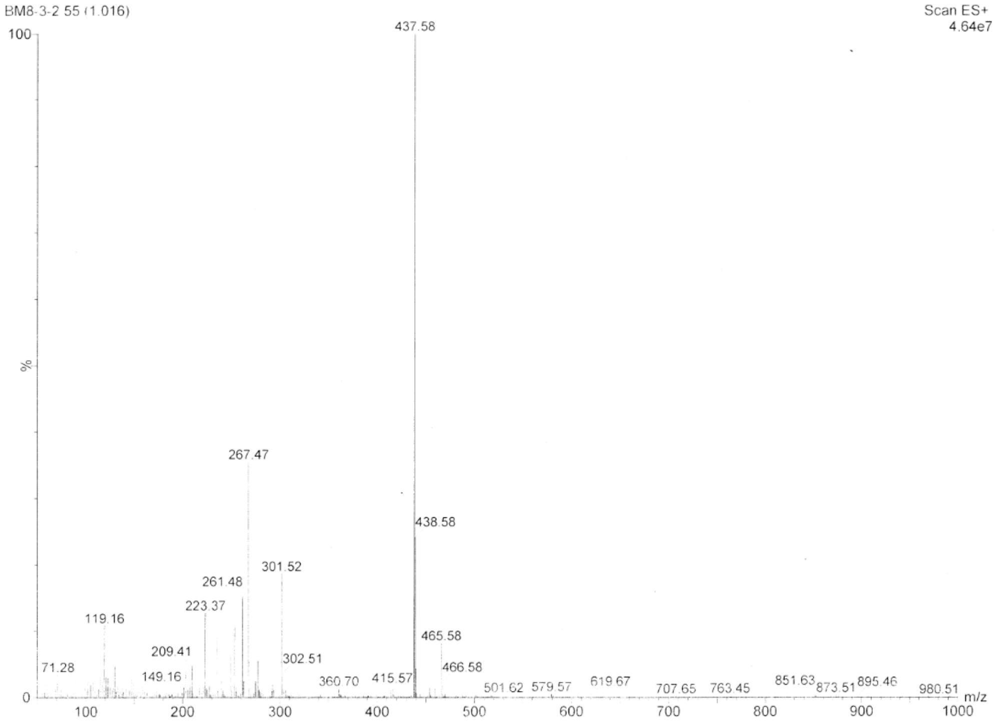

Figure S16. (+)-ESI-MS spectrum of **4**

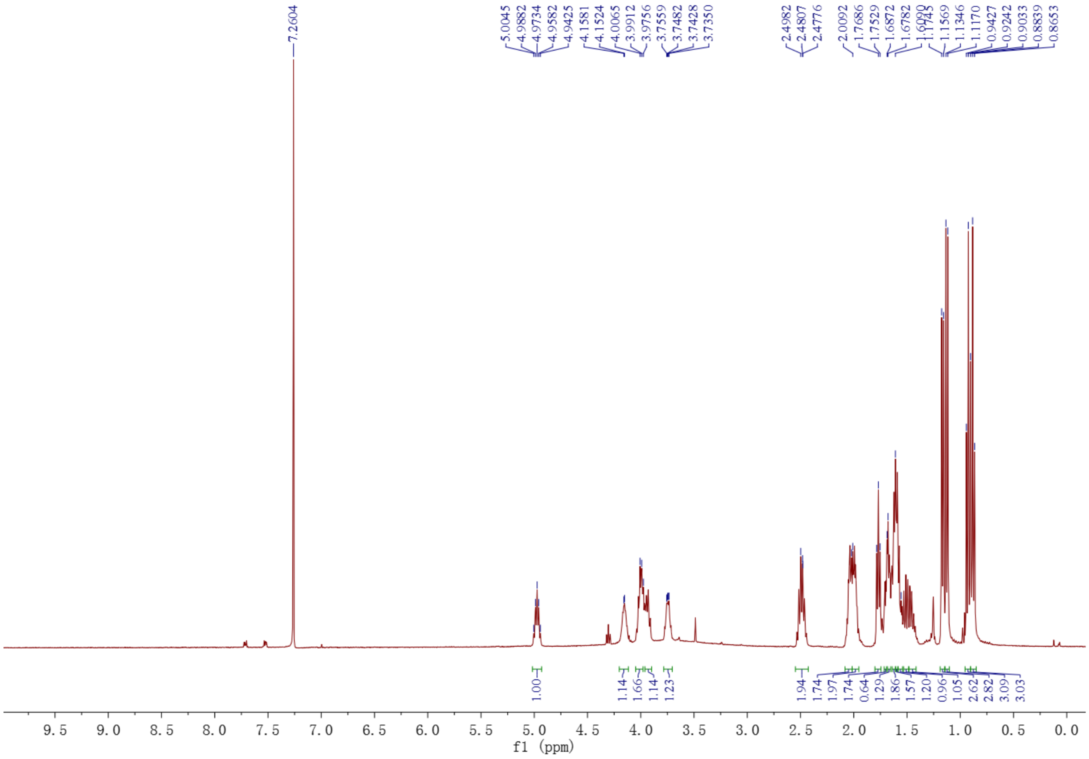

Figure S17. ^{1}H NMR spectrum of **5** in $CDCl_3$

© 2019 ACG Publications. All rights reserved.

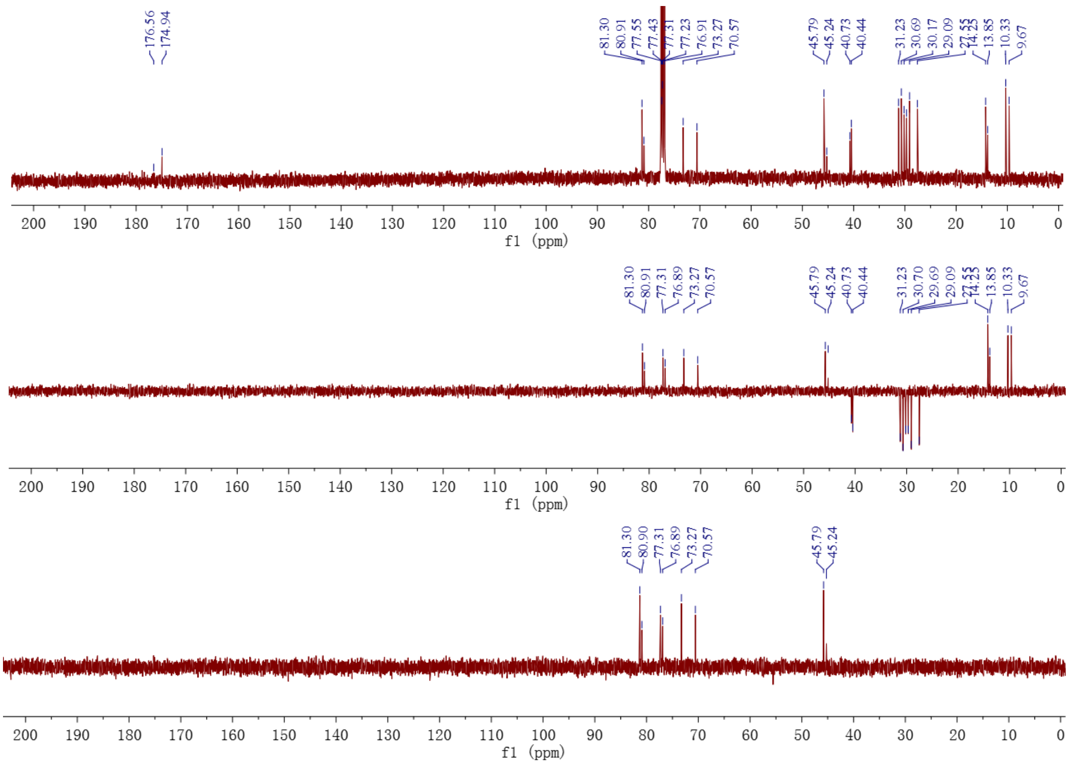

Figure S18. ^{13}C NMR and DEPT spectrum of **5** in $CDCl_3$

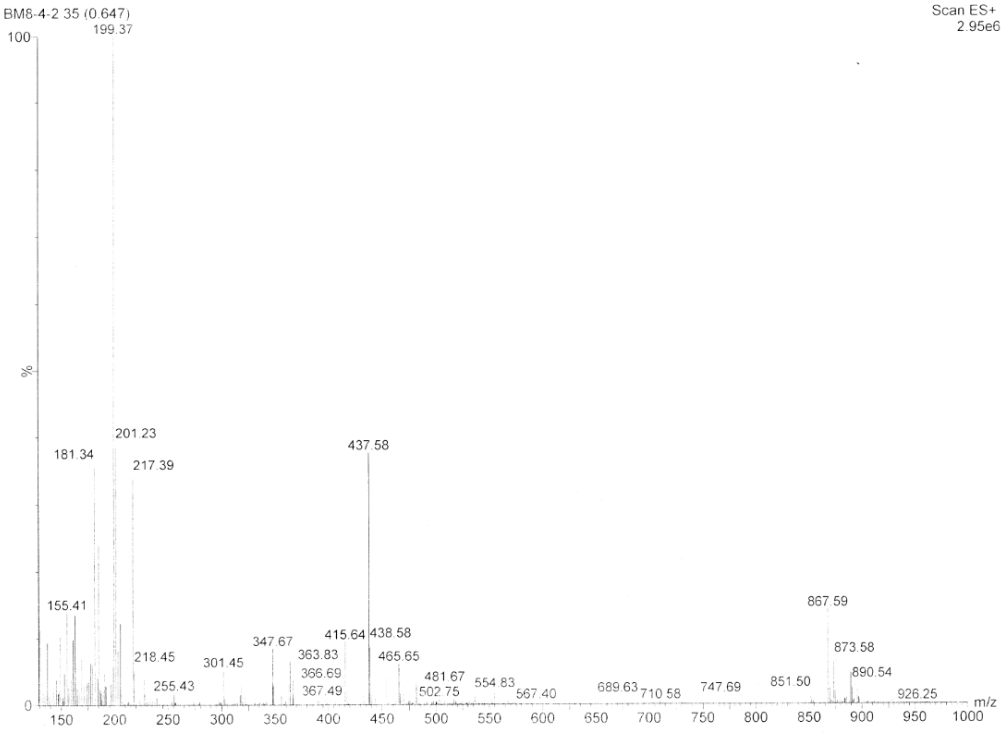

Figure S19. (+)-ESI-MS spectrum of **5**

© 2019 ACG Publications. All rights reserved.

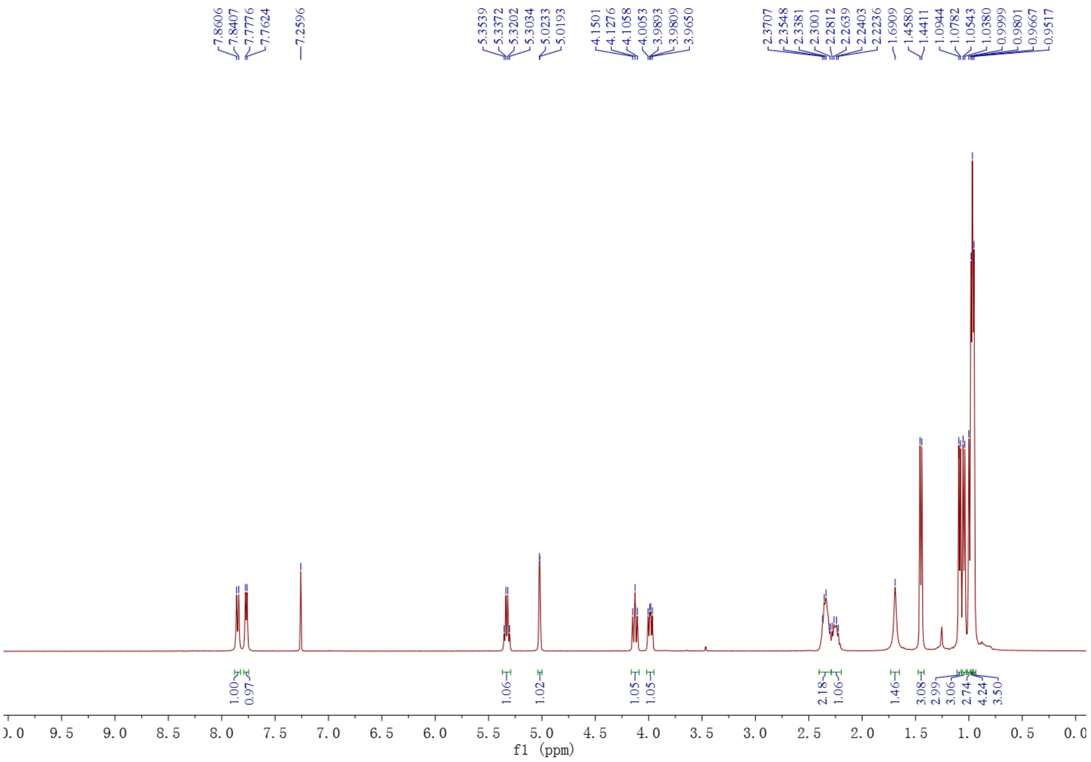

Figure S20. ^{1}H NMR spectrum of **6** in $CDCl_3$

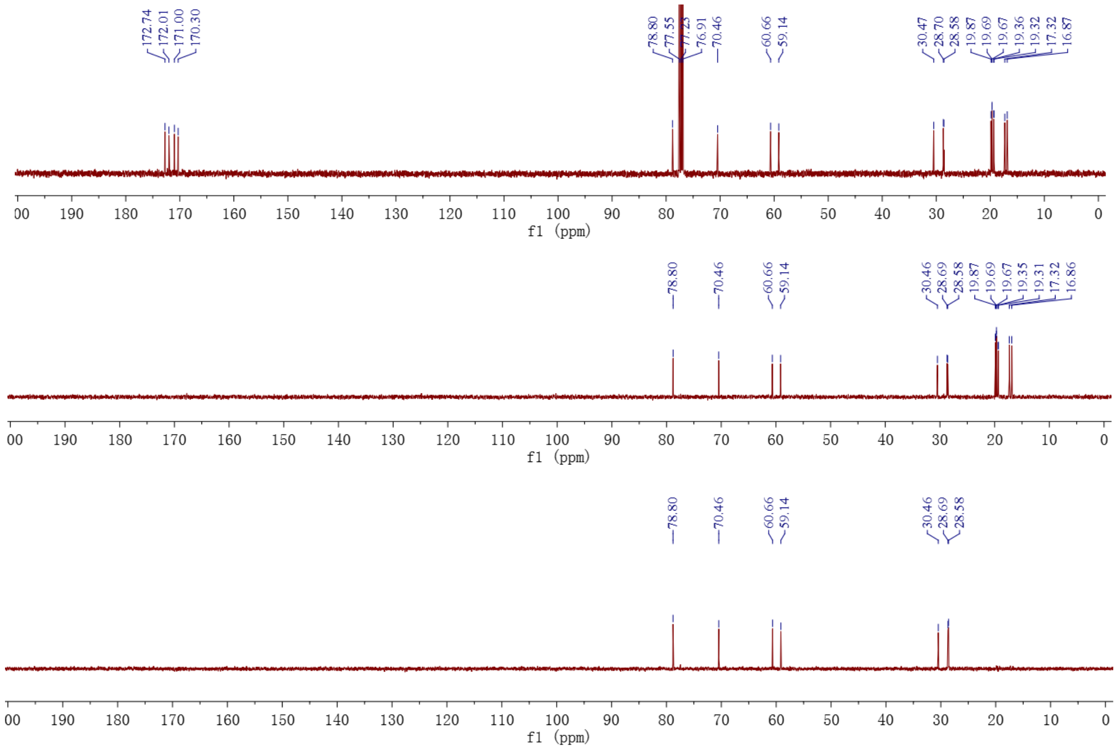

Figure S21. ^{13}C NMR and DEPT spectrum of **6** in $CDCl_3$

© 2019 ACG Publications. All rights reserved.

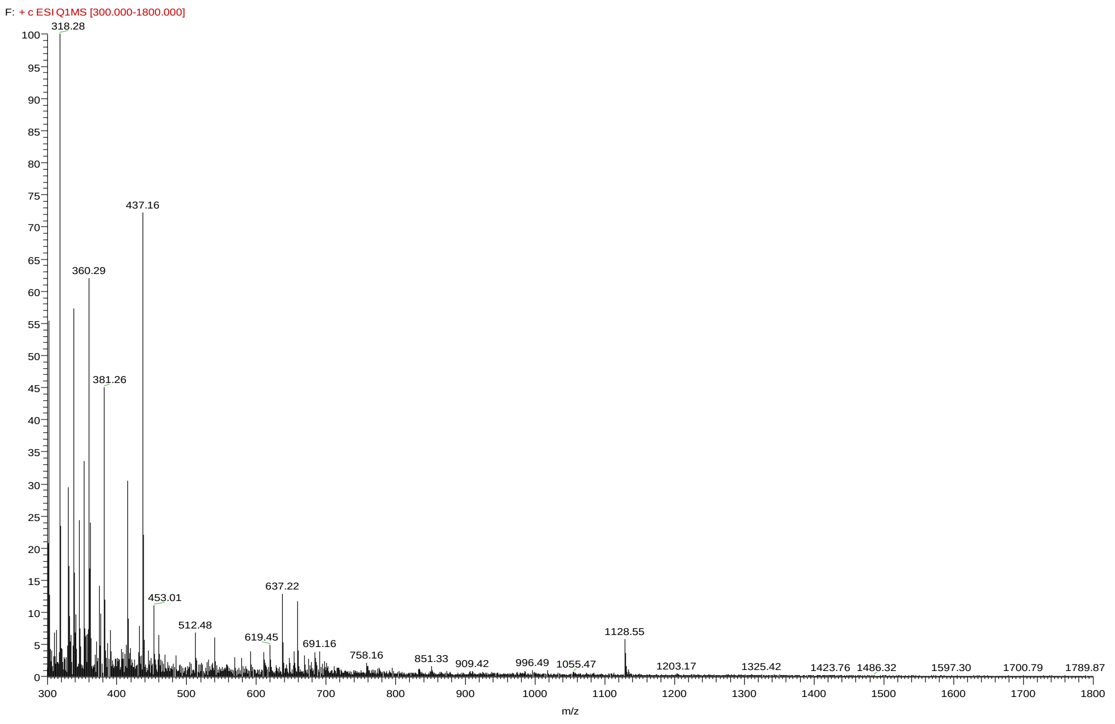

Figure S22. **(+)**-ESI-MS spectrum of **6**

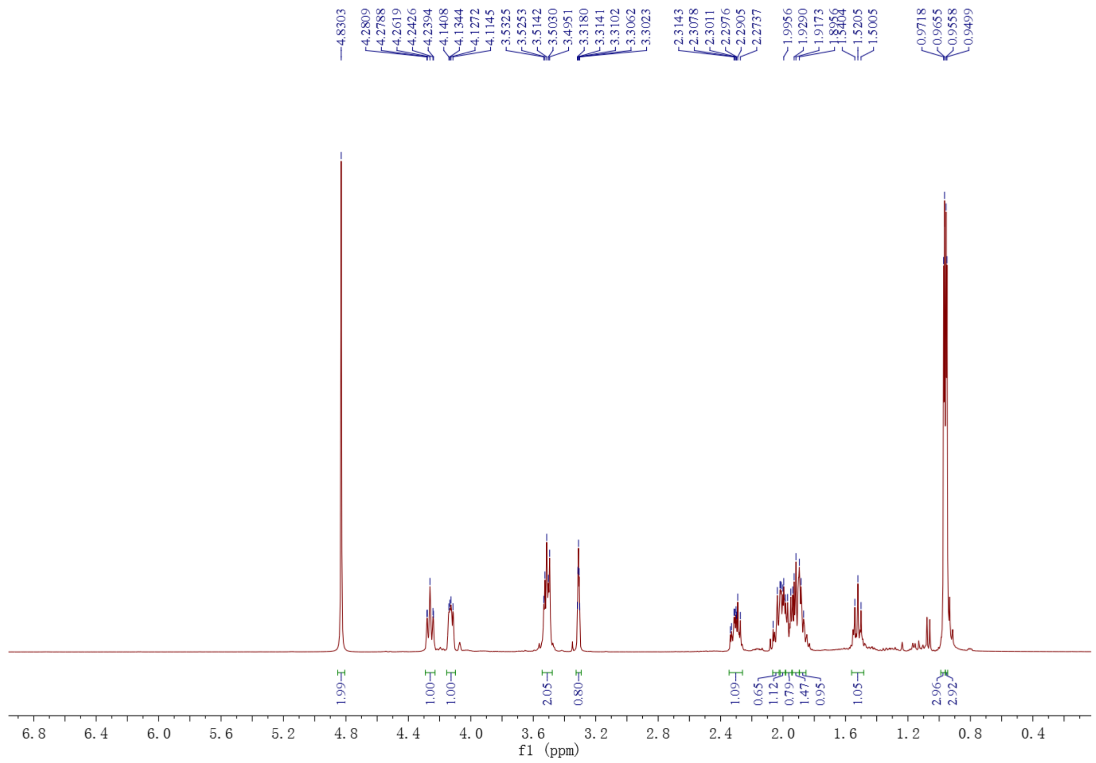

Figure S23. ^{1}H NMR spectrum of **7** in CD_3OD

© 2019 ACG Publications. All rights reserved.

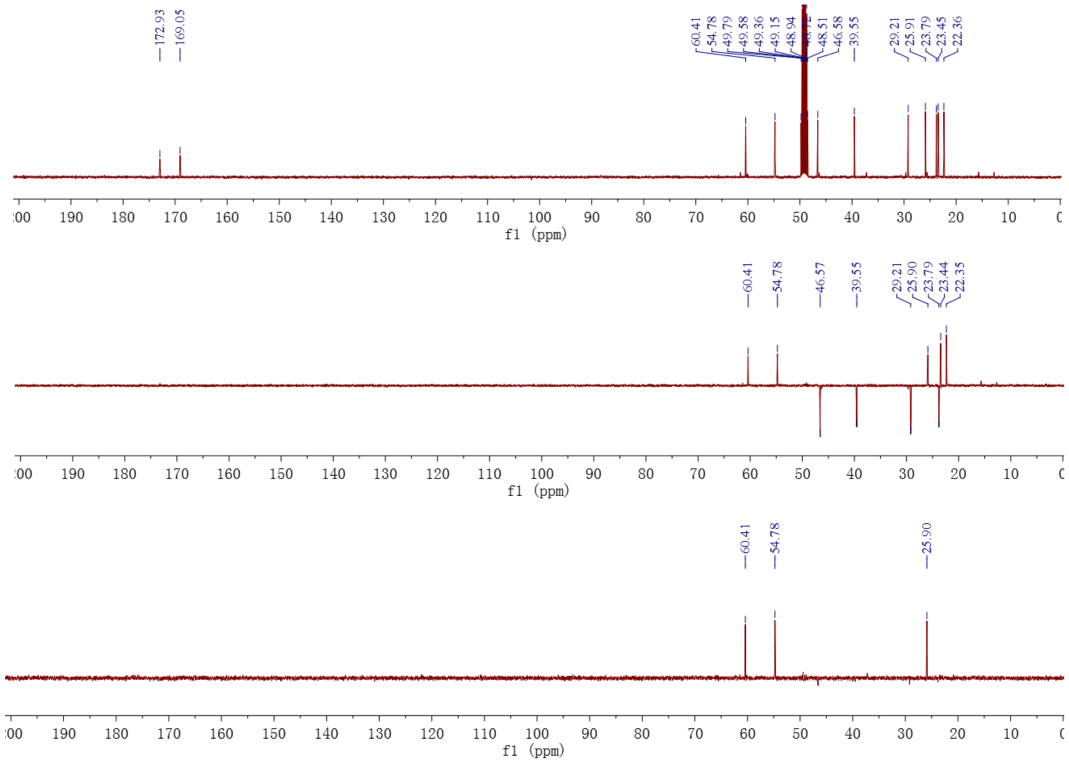

Figure S24. ^{13}C NMR and DEPT spectrum of **7** in CD_3OD

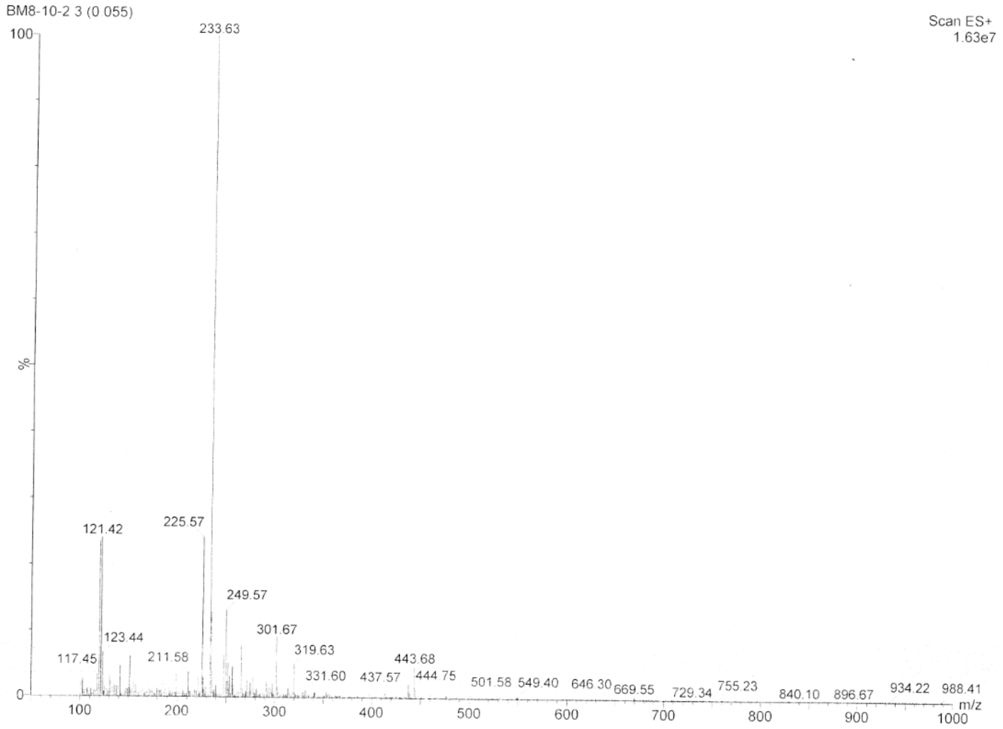

Figure S25. **(+)**-ESI-MS spectrum of **7**

© 2019 ACG Publications. All rights reserved.

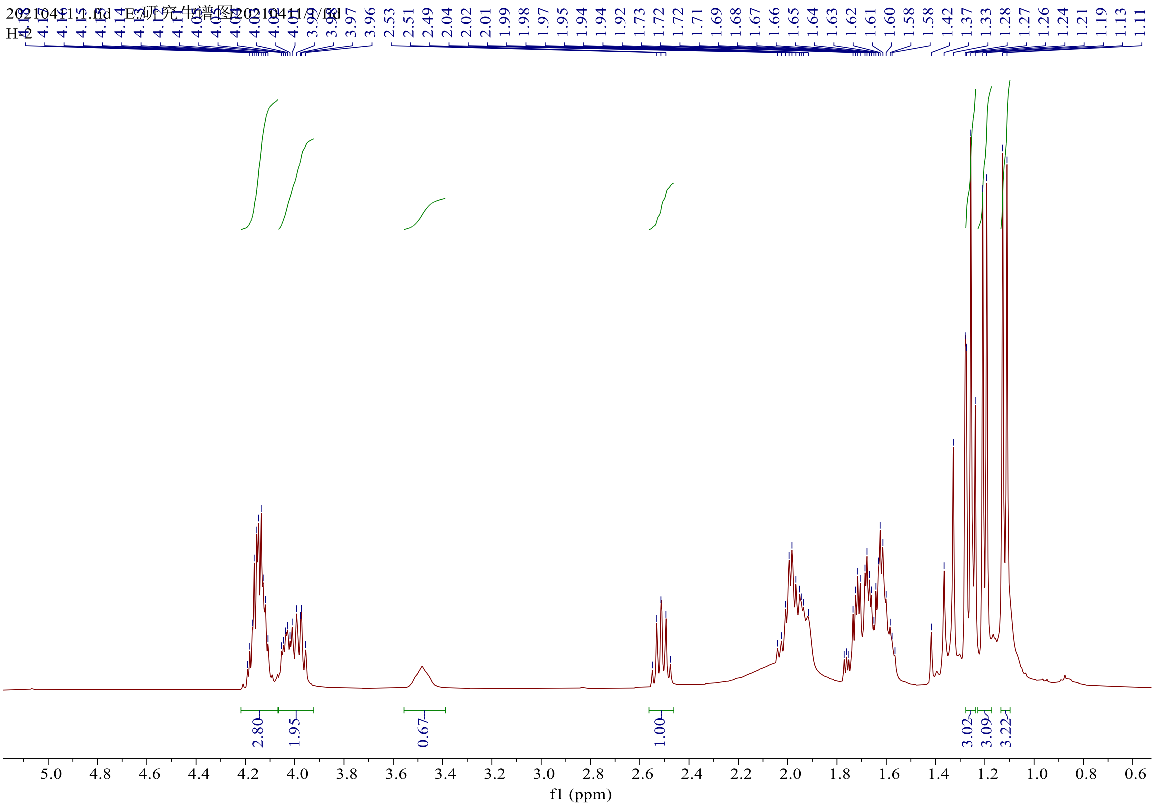

Figure S26. ^{1}H NMR spectrum of **8** in $CDCl_3$

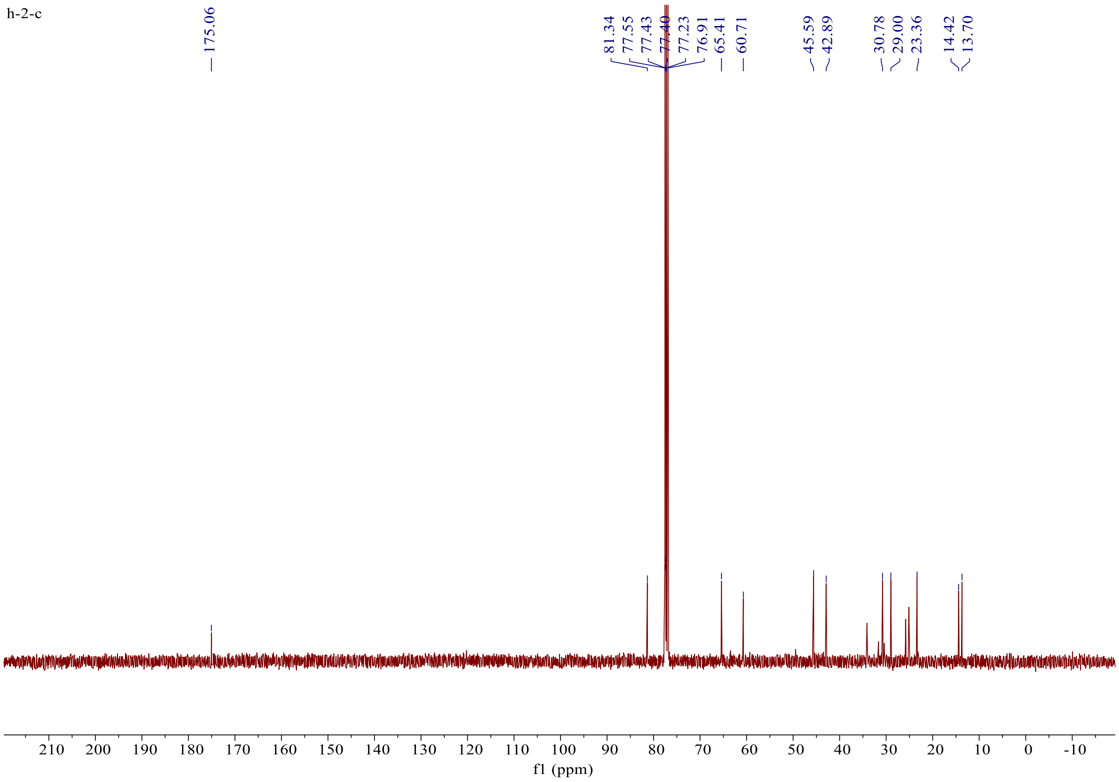

Figure S27. ^{13}C NMR spectrum of **8** in $CDCl_3$

© 2019 ACG Publications. All rights reserved.

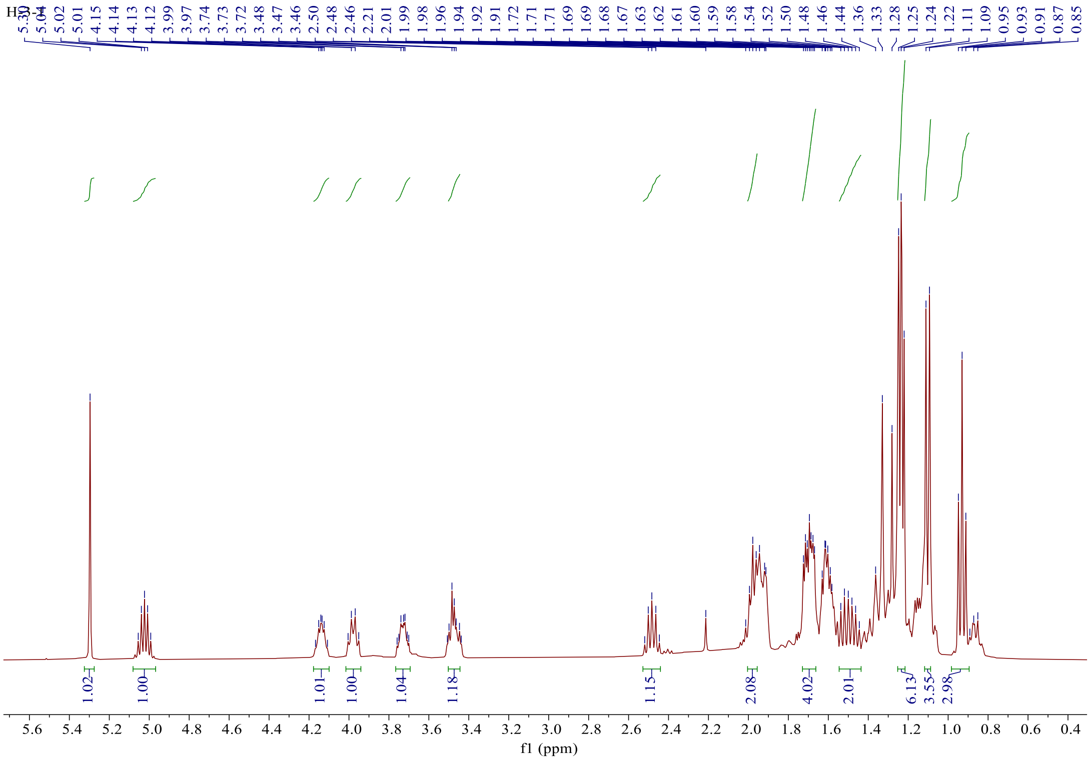

Figure S28. ^{1}H NMR spectrum of **9** in $CDCl_3$

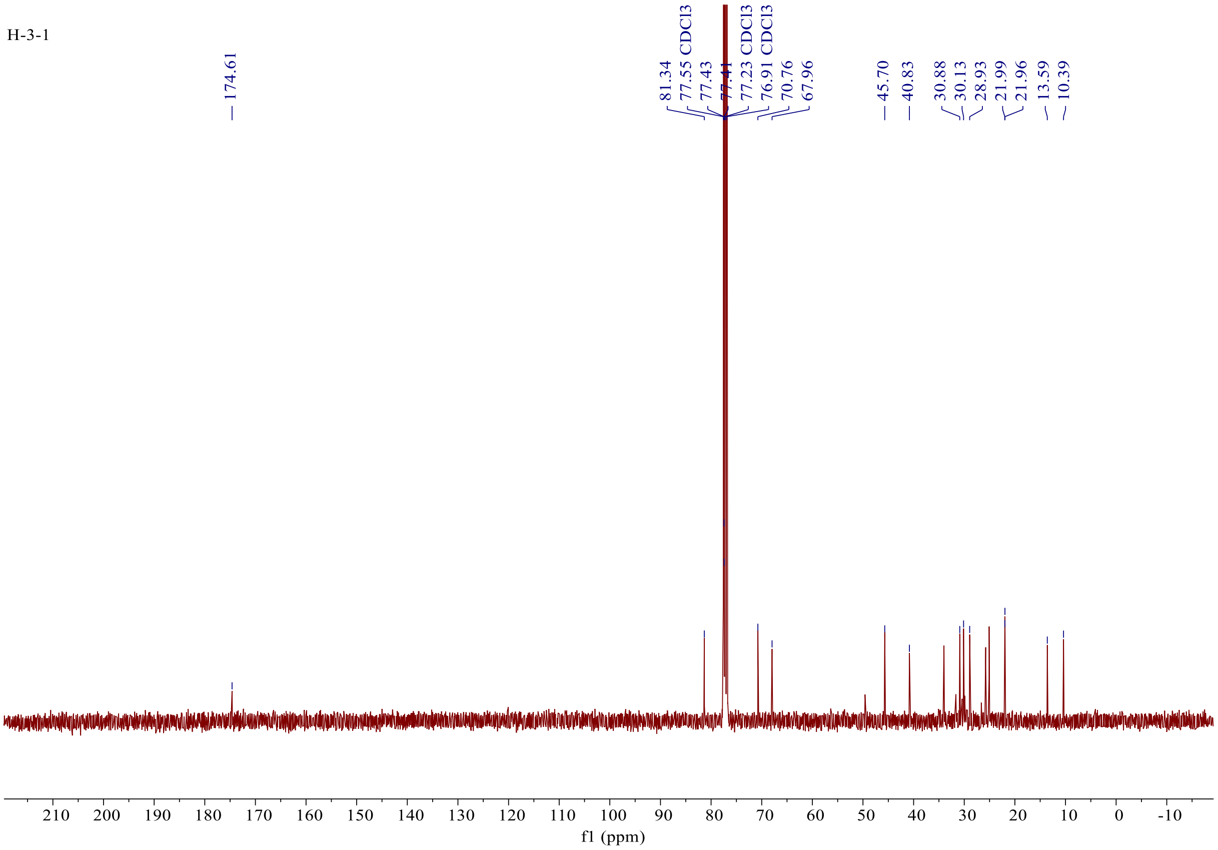

Figure S29. ^{13}C NMR spectrum of **9** in $CDCl_3$

© 2019 ACG Publications. All rights reserved.

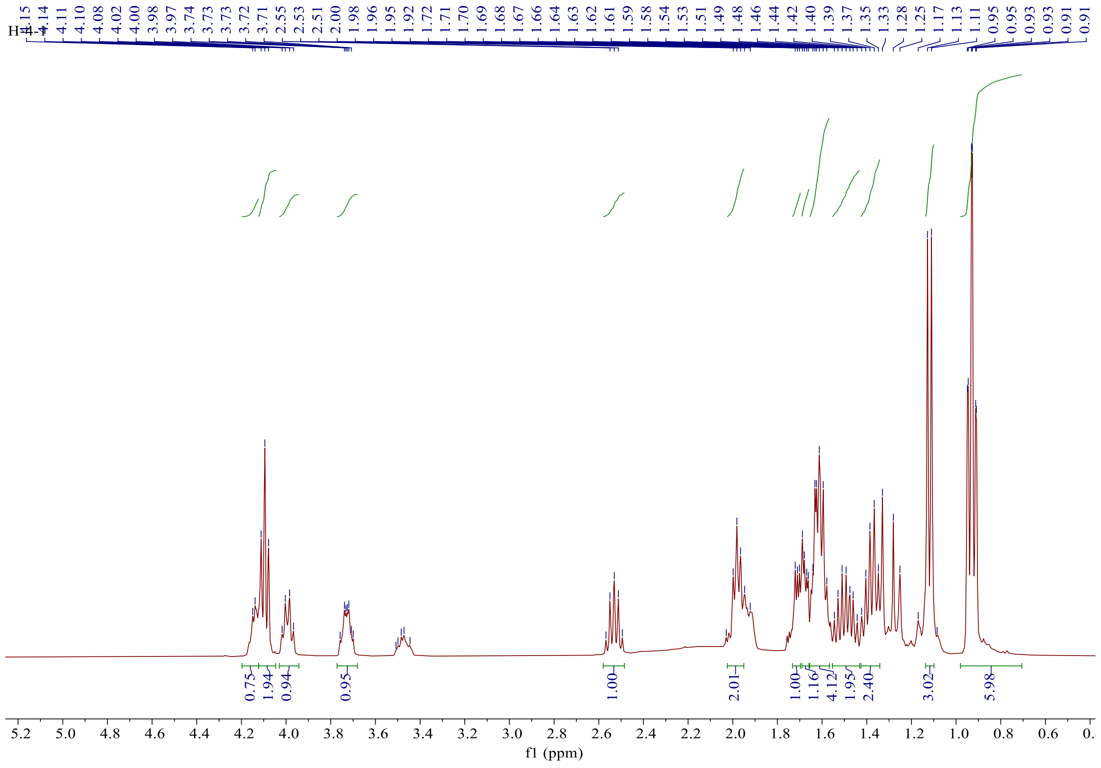

Figure S30. ^{1}H NMR spectrum of **10** in $CDCl_3$

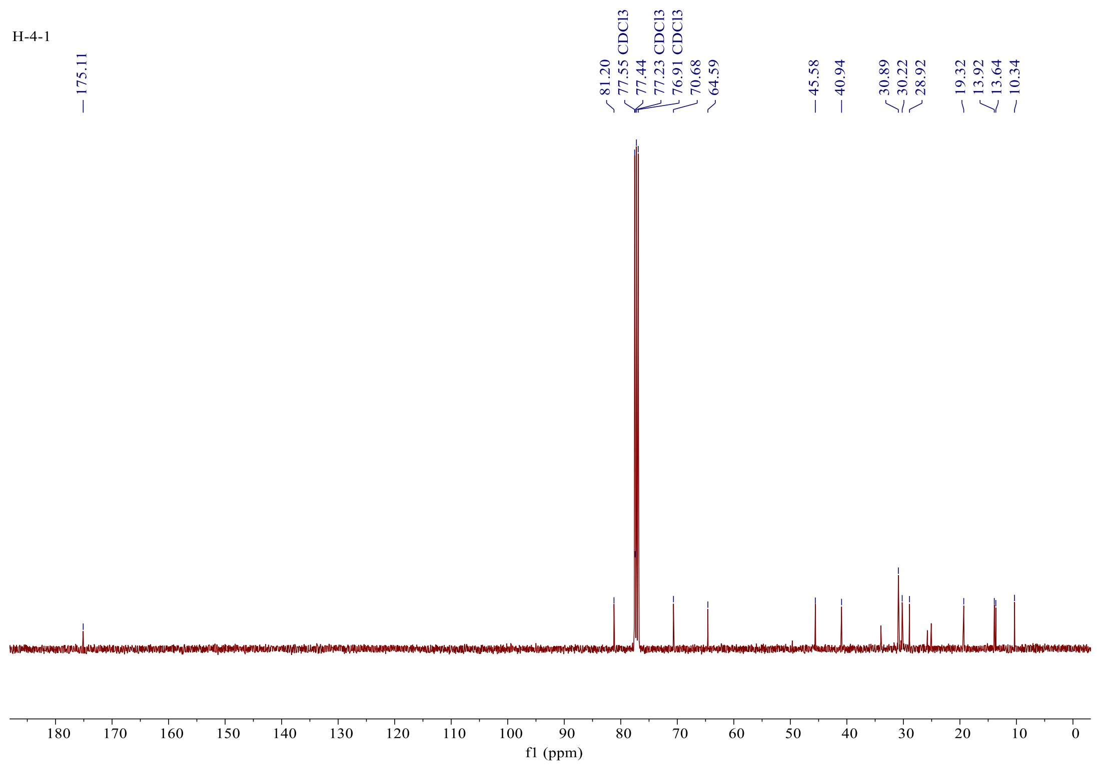

Figure S31. ^{13}C NMR spectrum of **10** in $CDCl_3$

© 2019 ACG Publications. All rights reserved.

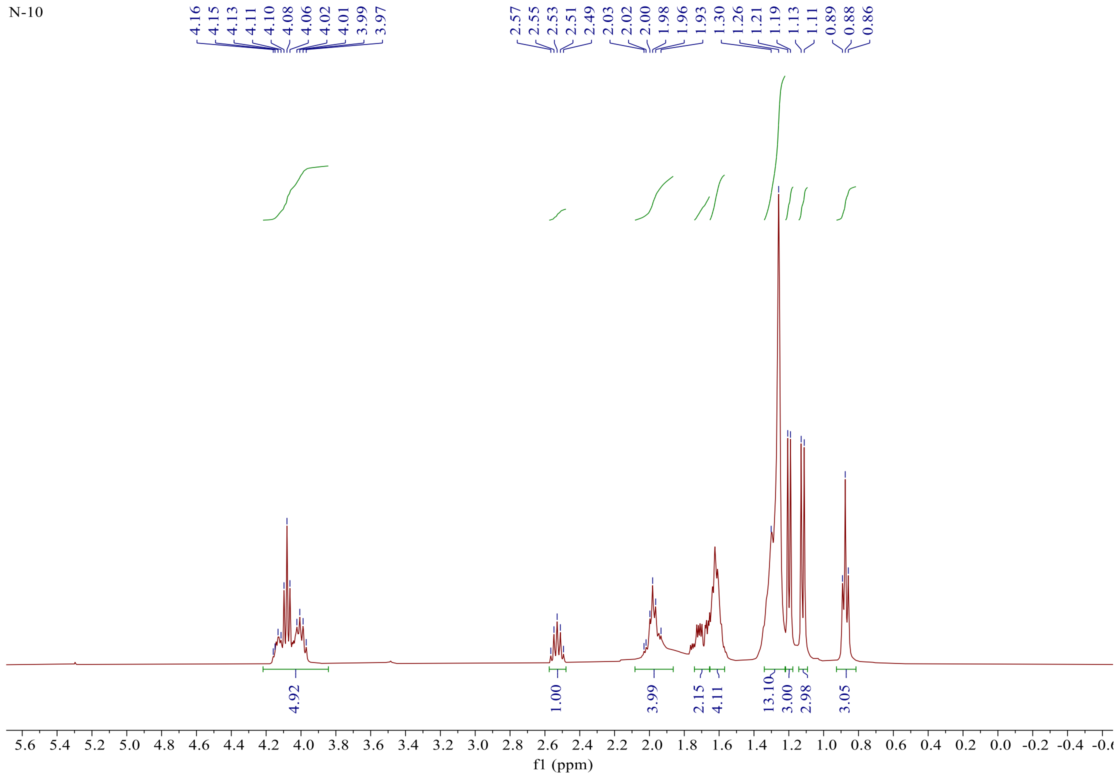

Figure S32. ^{1}H NMR spectrum of **11** in $CDCl_3$

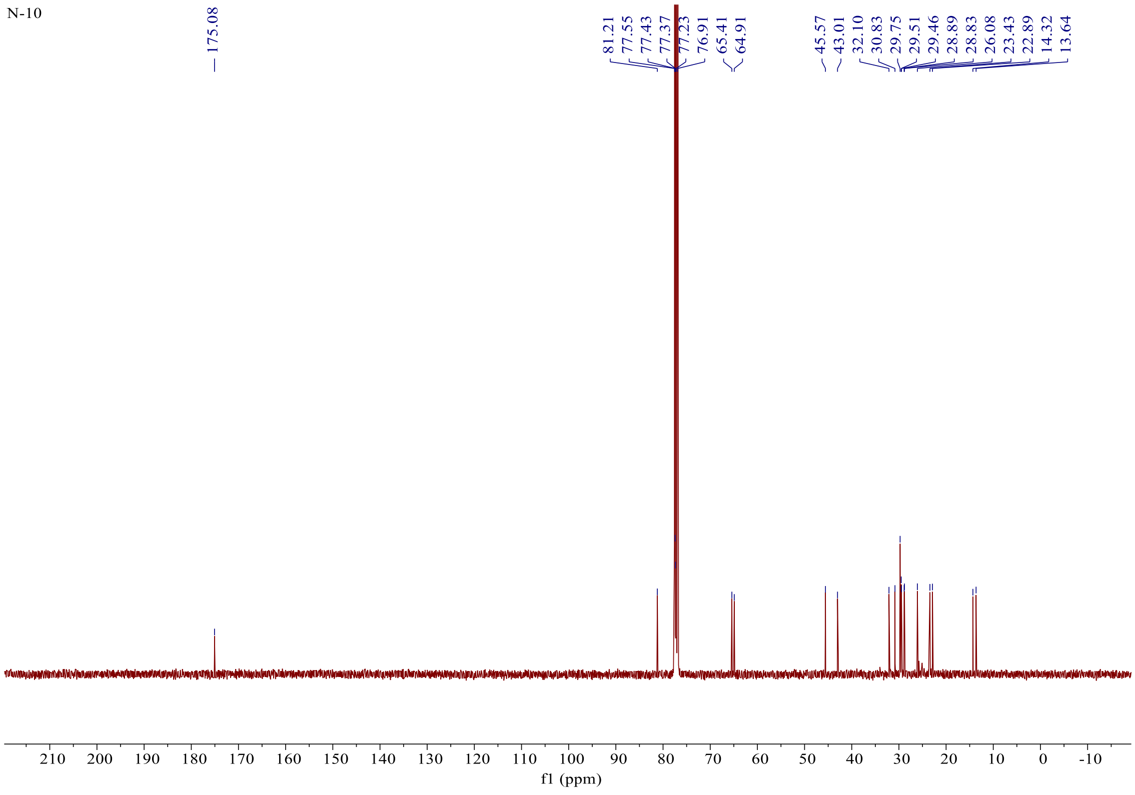

Figure S33. ^{13}C NMR spectrum of **11** in $CDCl_3$

© 2019 ACG Publications. All rights reserved.

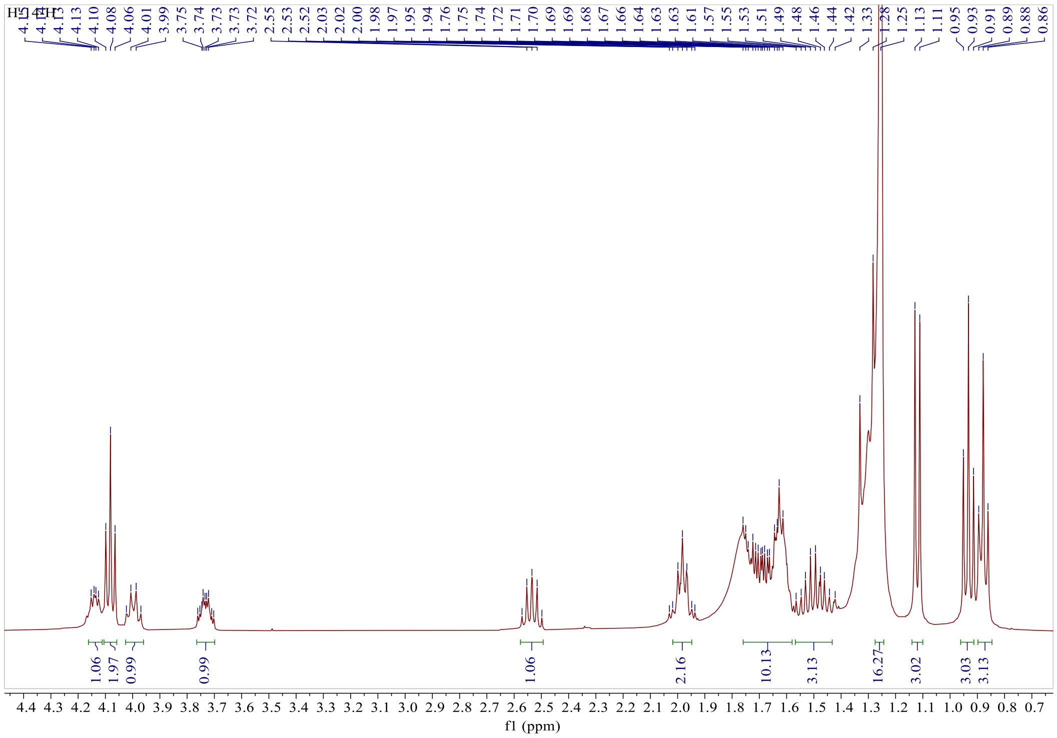

Figure S34. ^{1}H NMR spectrum of **12** in $CDCl_3$

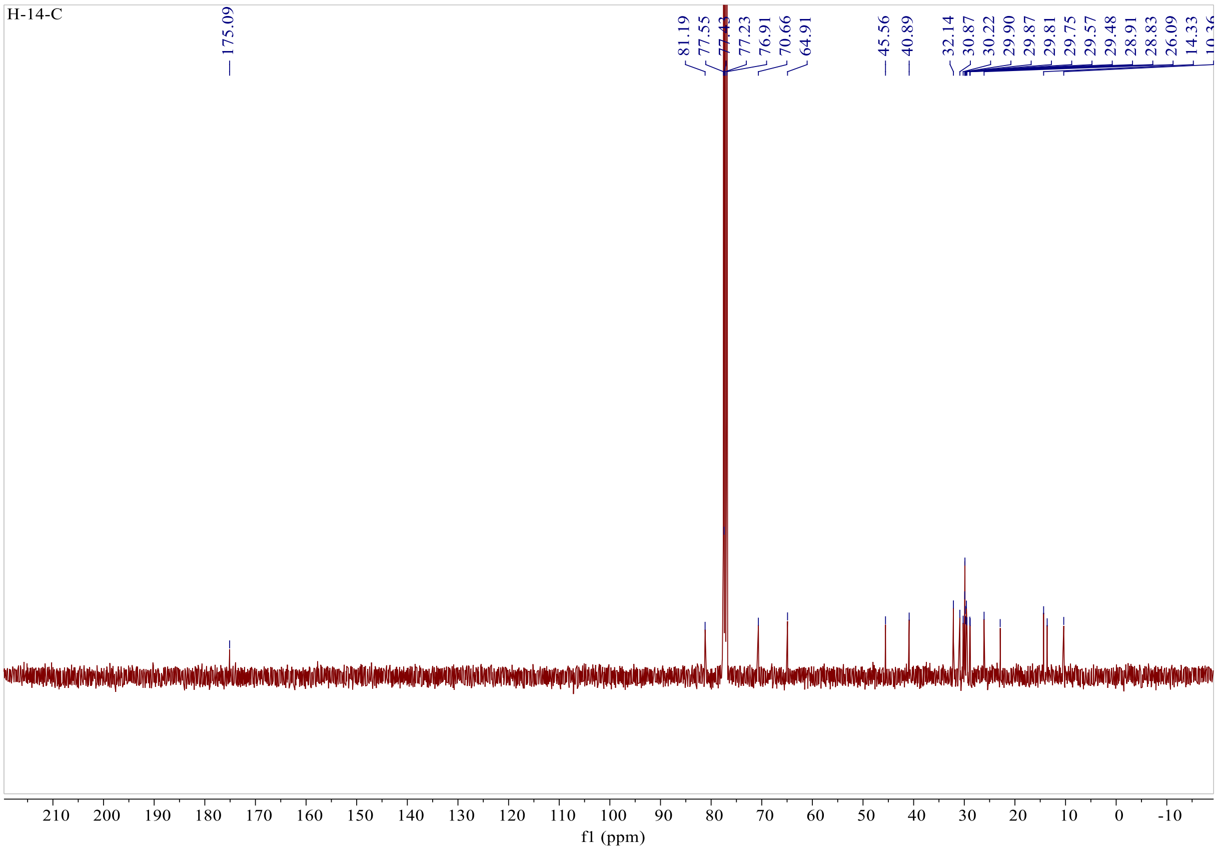

Figure S35. ^{13}C NMR spectrum of **12** in $CDCl_3$

© 2019 ACG Publications. All rights reserved.

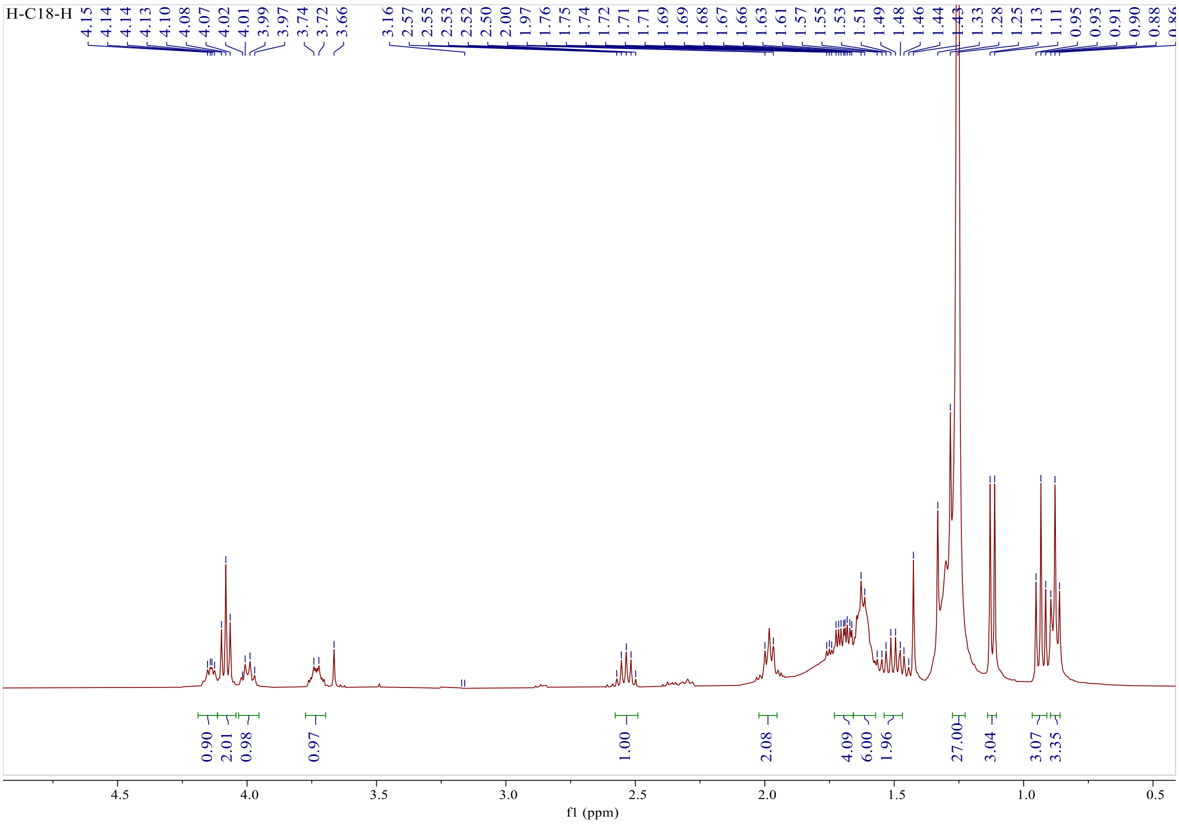

Figure S36. ^{1}H NMR spectrum of **13** in $CDCl_3$

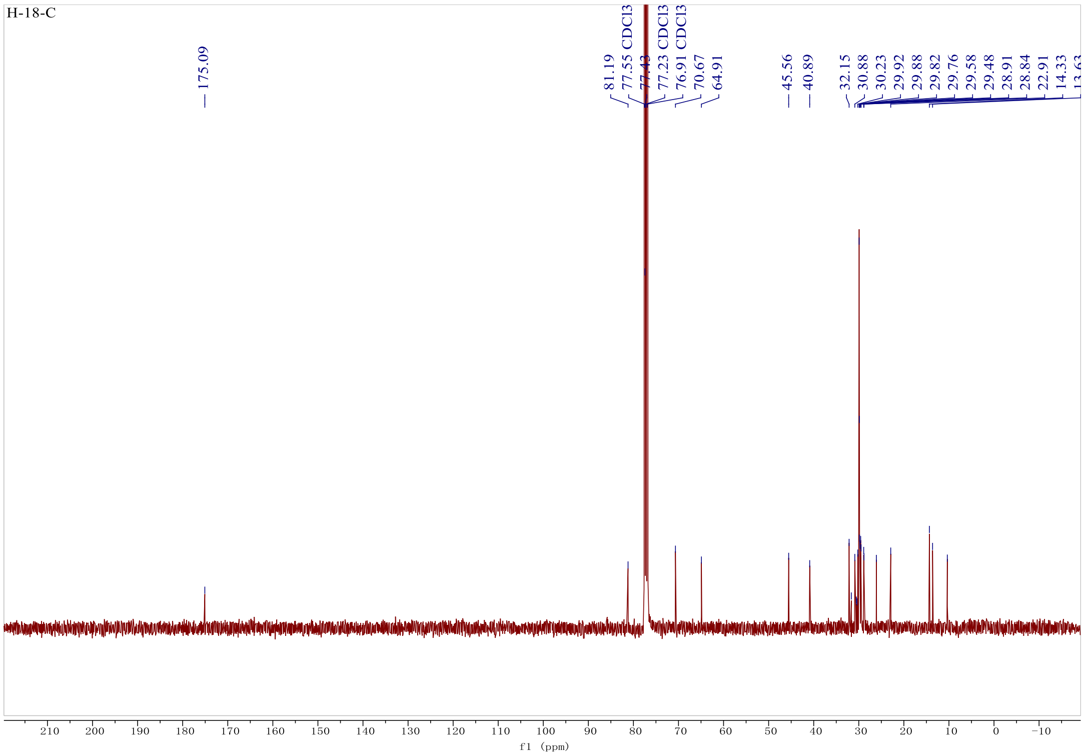

Figure S37. ^{13}C NMR spectrum of **13** in $CDCl_3$

© 2019 ACG Publications. All rights reserved.

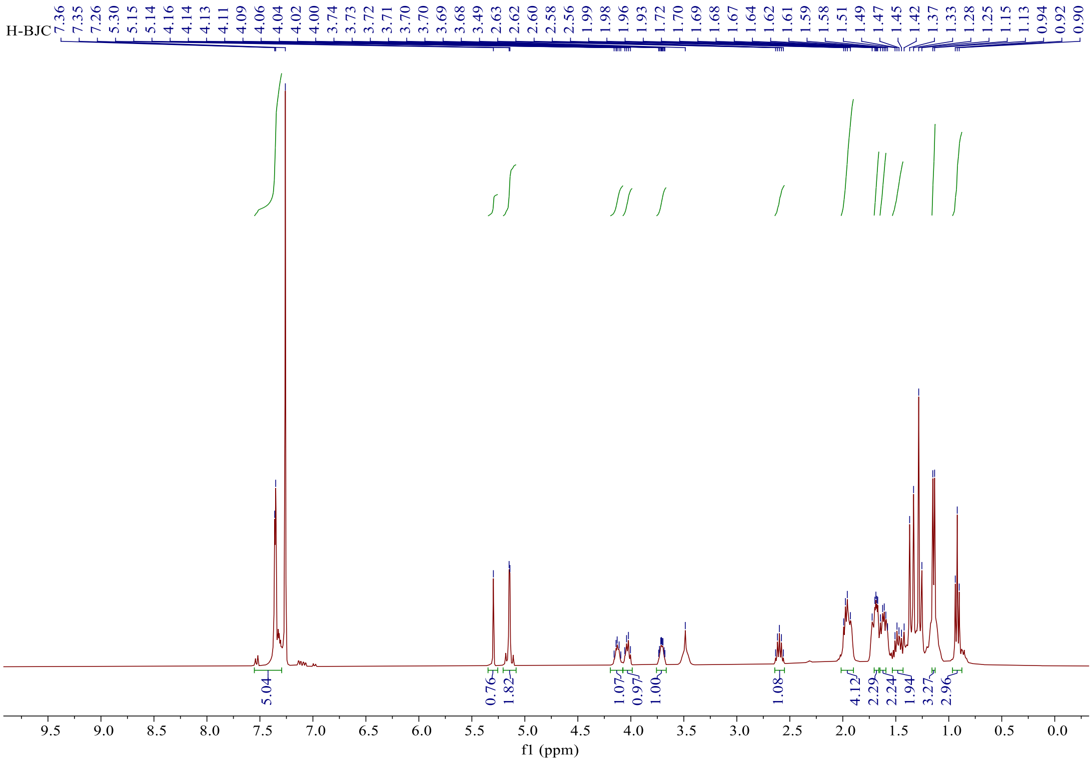

Figure S38. ^{1}H NMR spectrum of **14** in $CDCl_3$

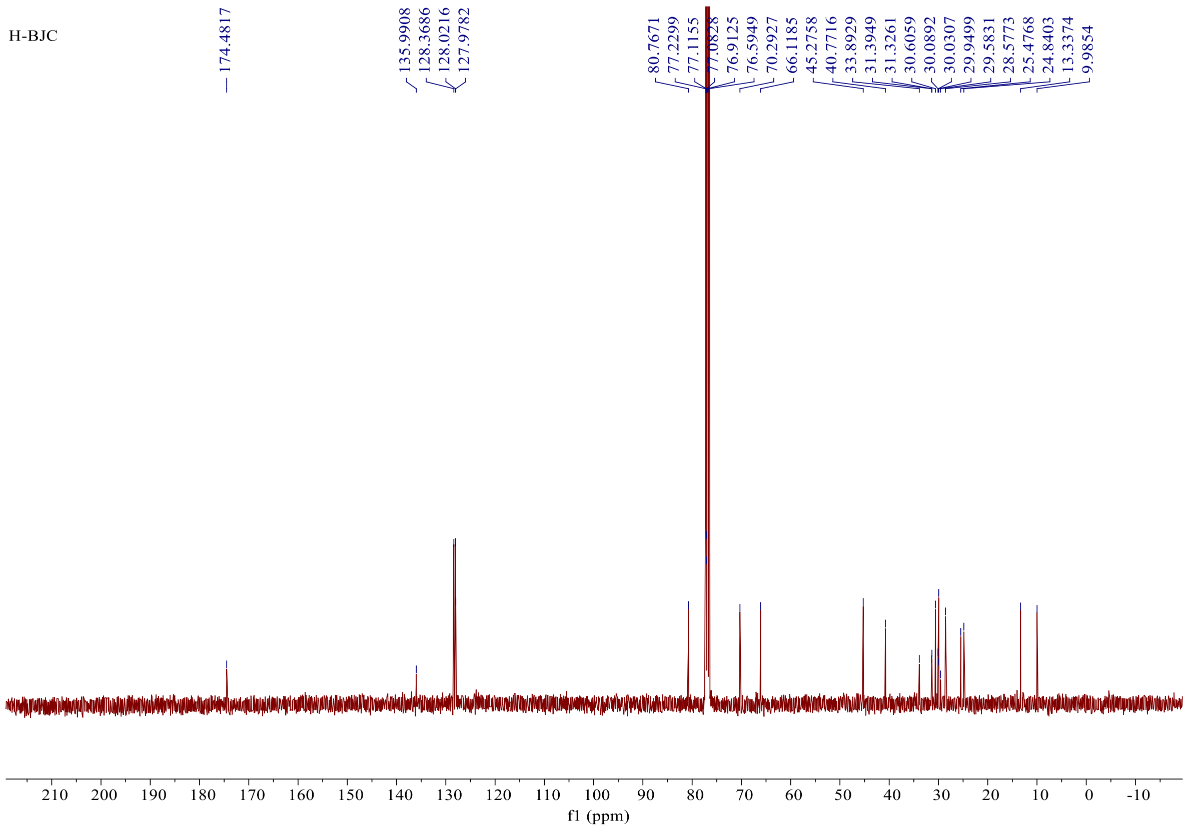

Figure S39. ^{13}C NMR spectrum of **14** in $CDCl_3$

© 2019 ACG Publications. All rights reserved.

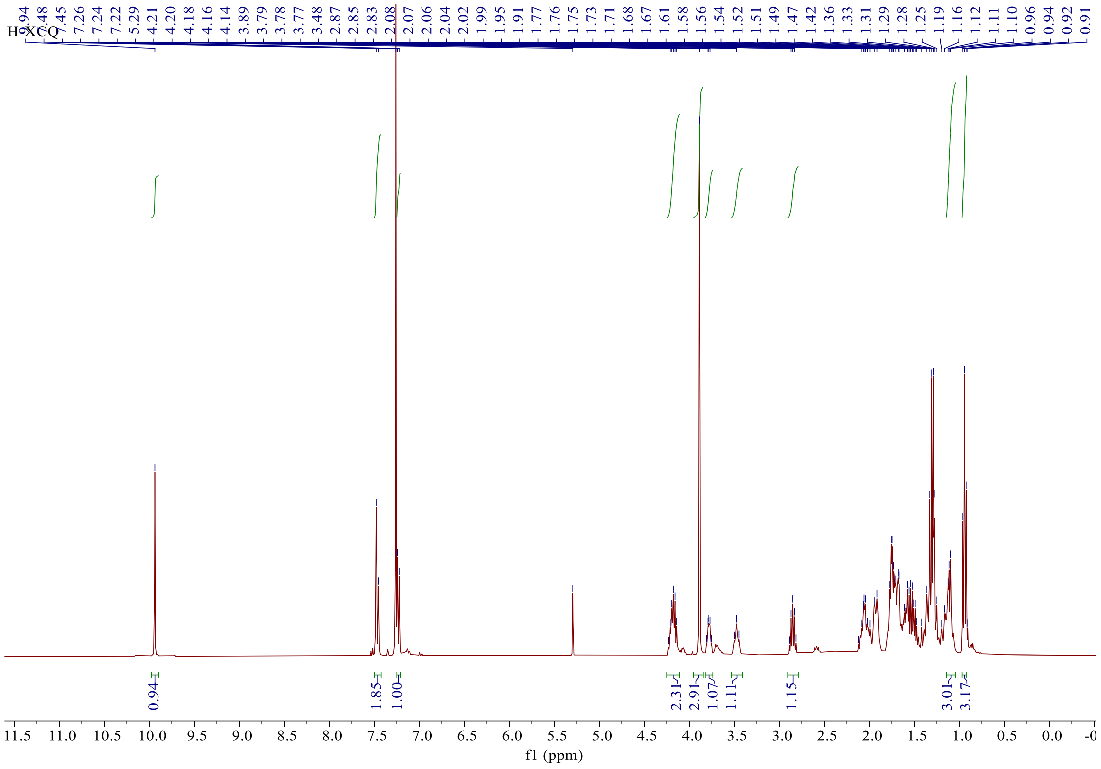

Figure S40. ^{1}H NMR spectrum of **15** in $CDCl_3$

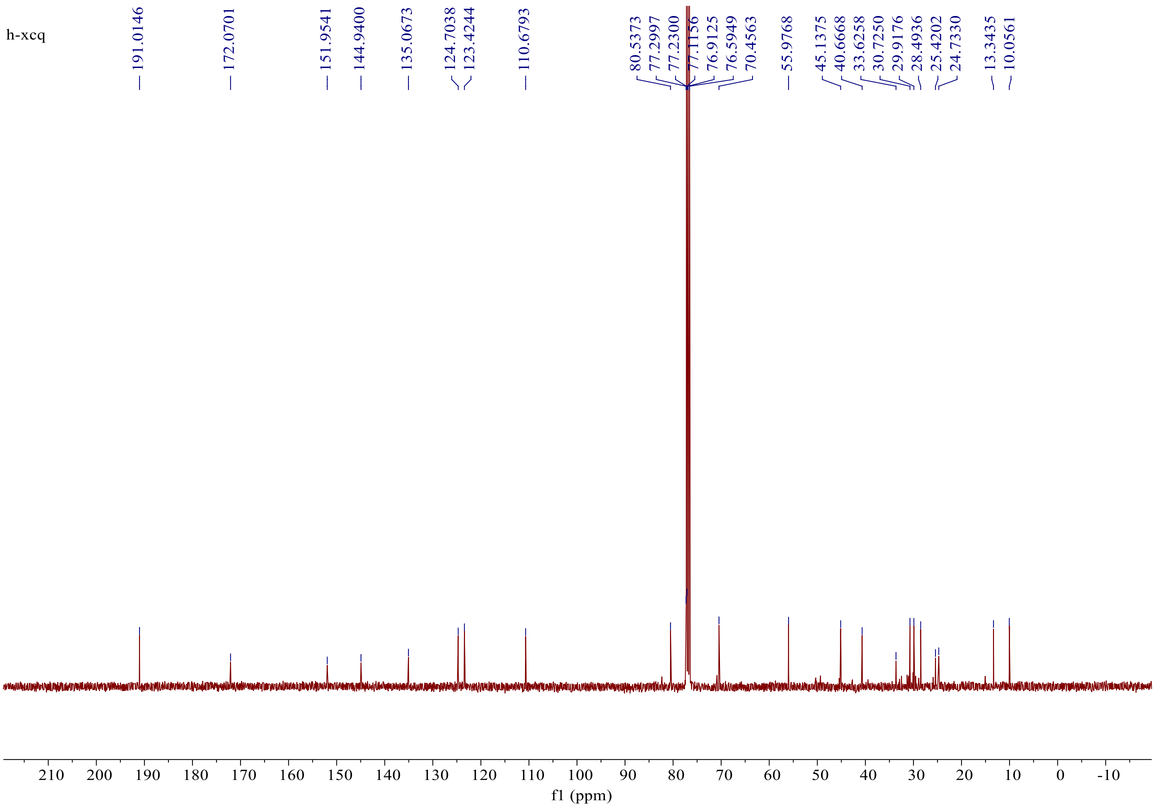

Figure S41. ^{13}C NMR spectrum of **15** in $CDCl_3$

© 2019 ACG Publications. All rights reserved.

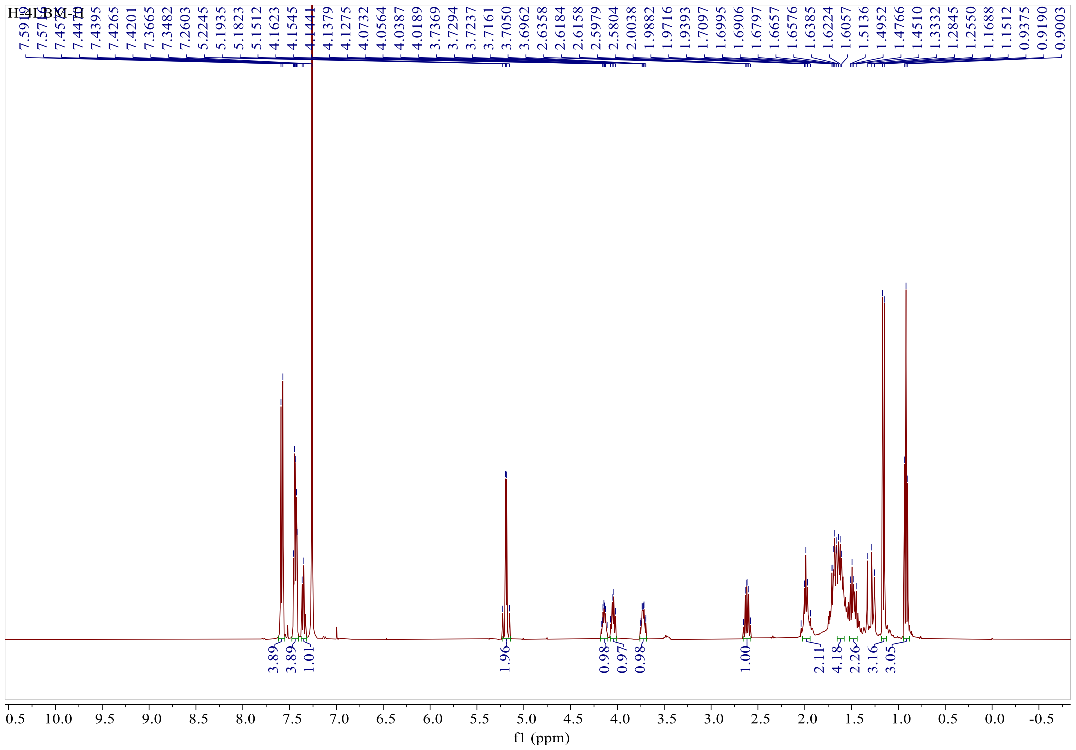

Figure S42. ^{1}H NMR spectrum of **16** in $CDCl_3$

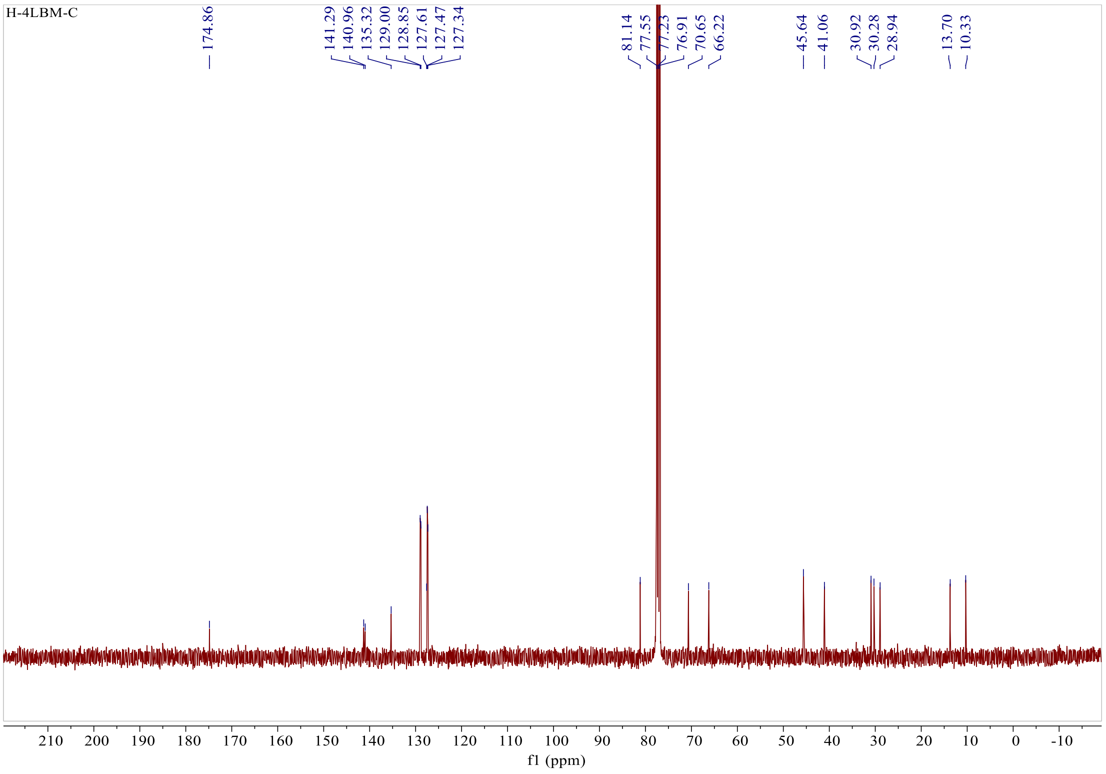

Figure S43. ^{13}C NMR spectrum of **16** in $CDCl_3$

© 2019 ACG Publications. All rights reserved.

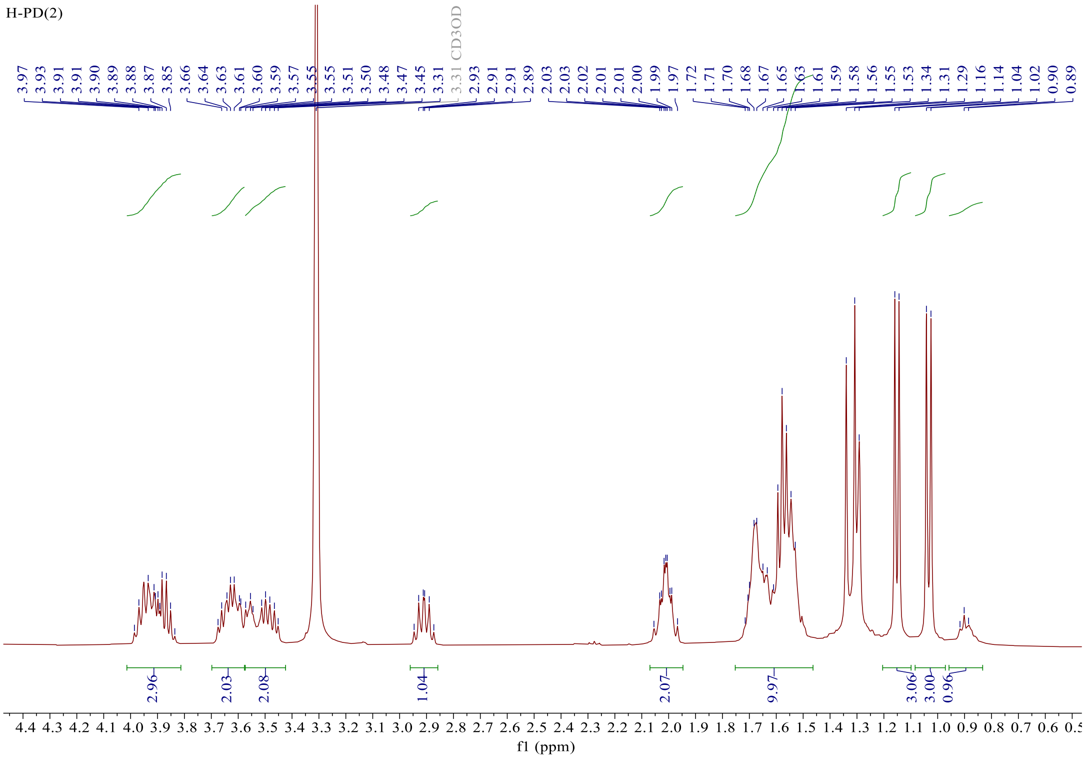

Figure S44. ^{1}H NMR spectrum of **17** in CD_3OD

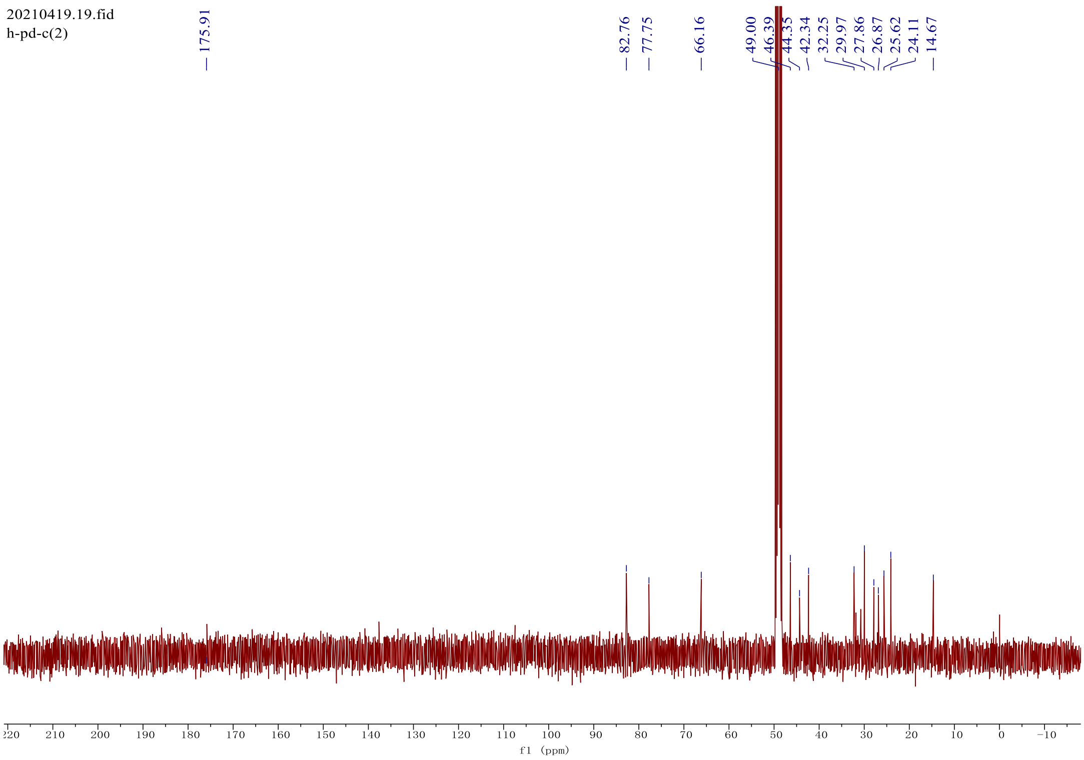

Figure S45. ^{13}C NMR spectrum of **17** in CD_3OD

© 2019 ACG Publications. All rights reserved.

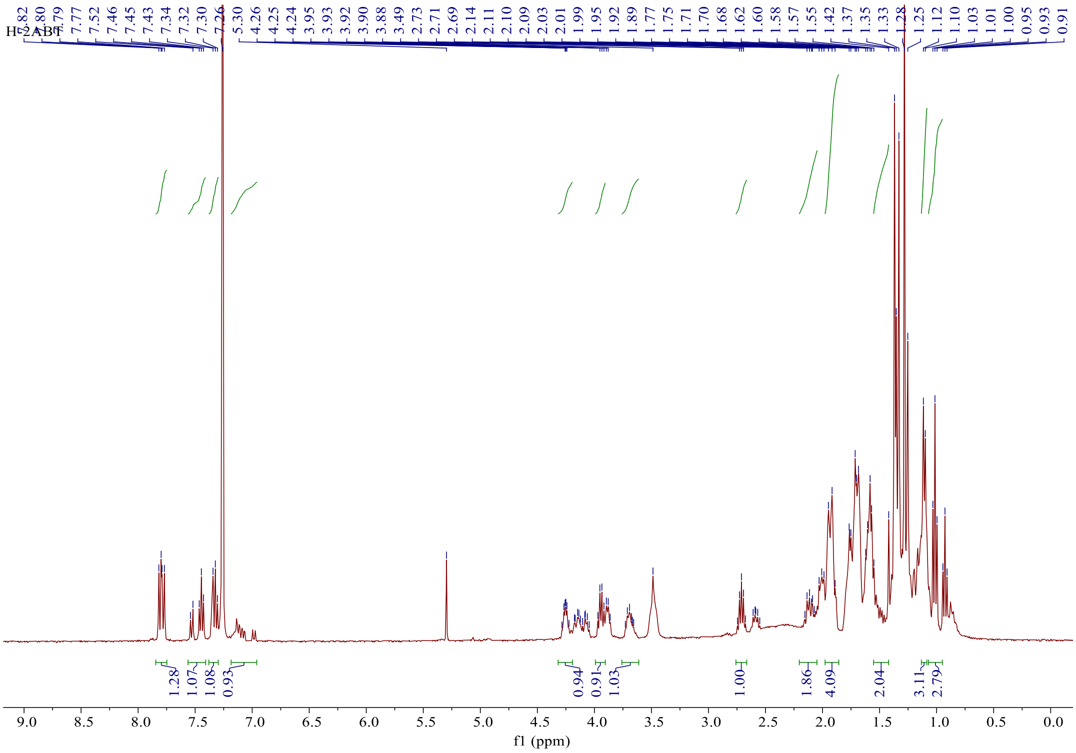

Figure S46. ^{1}H NMR spectrum of **19** in $CDCl_3$

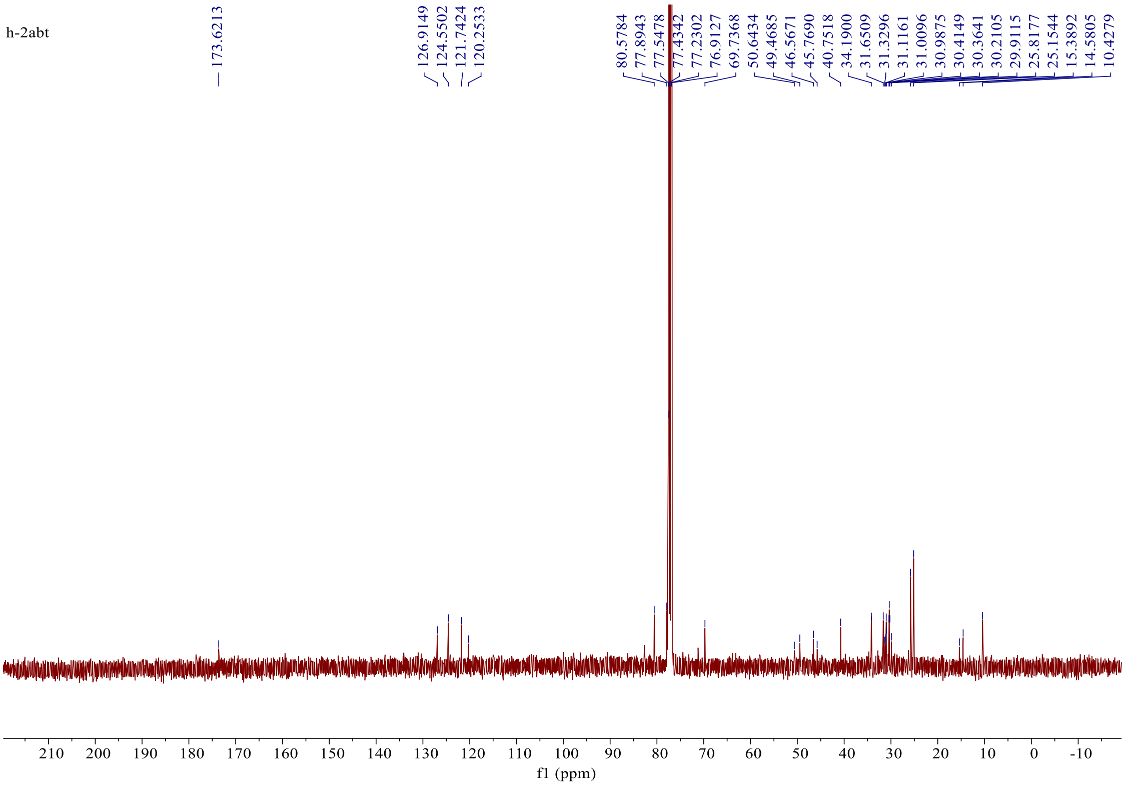

Figure S47. ^{13}C NMR spectrum of **19** in $CDCl_3$

© 2019 ACG Publications. All rights reserved.

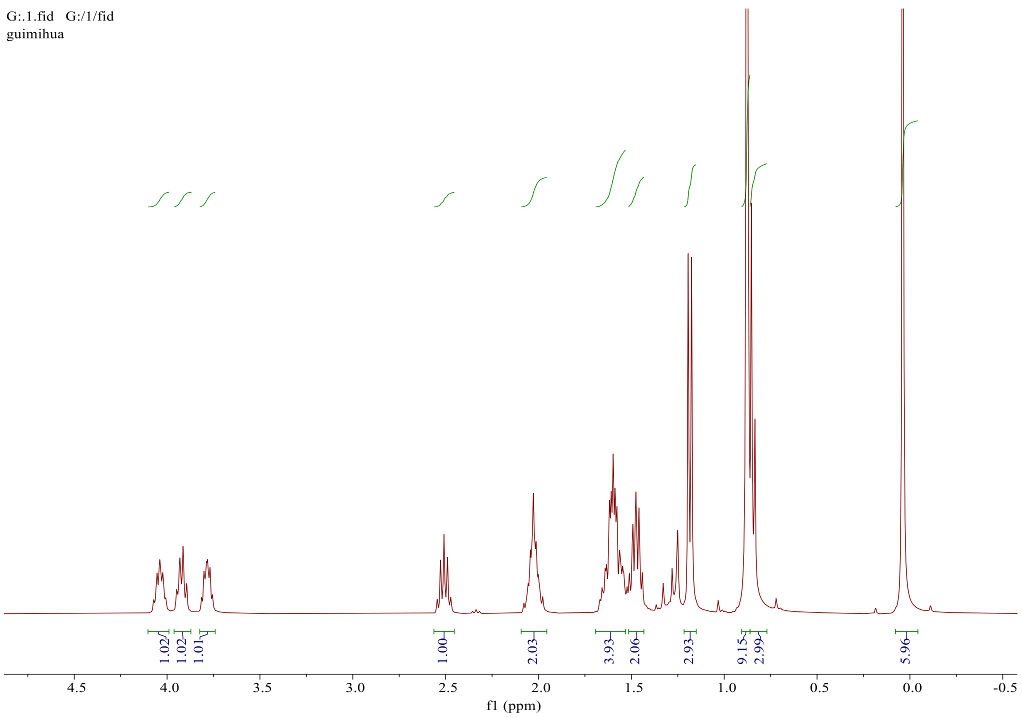

Figure S48. ^{1}H NMR spectrum of **20** in $CDCl_3$

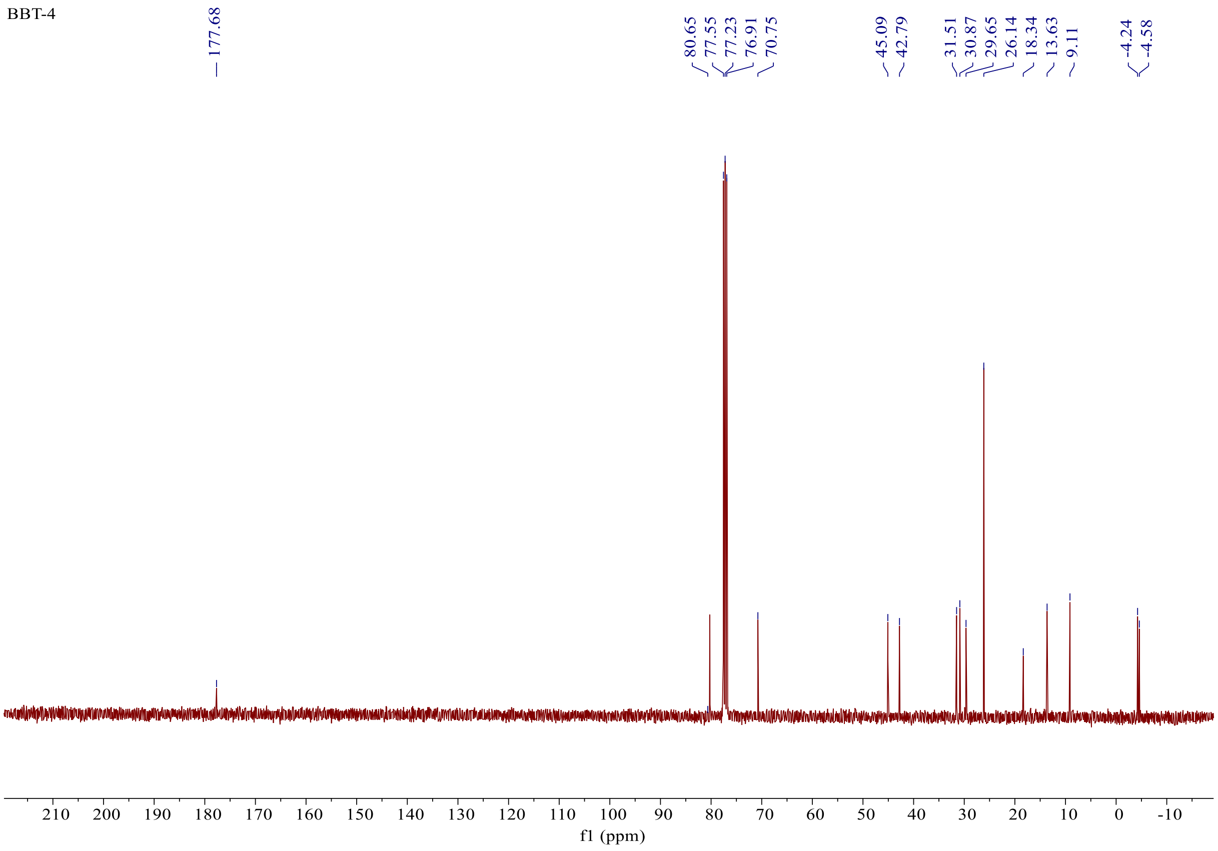

Figure S49. ^{13}C NMR spectrum of **20** in $CDCl_3$

© 2019 ACG Publications. All rights reserved.

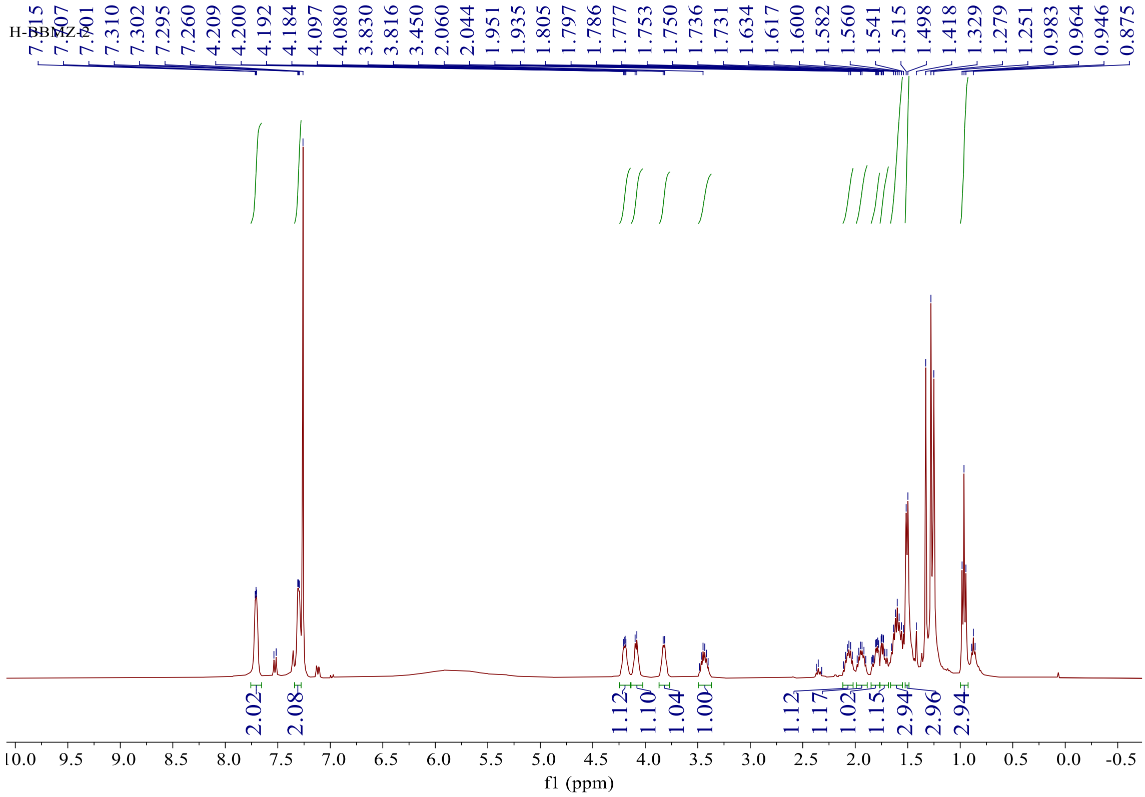

Figure S50. ^{1}H NMR spectrum of **21** in $CDCl_3$

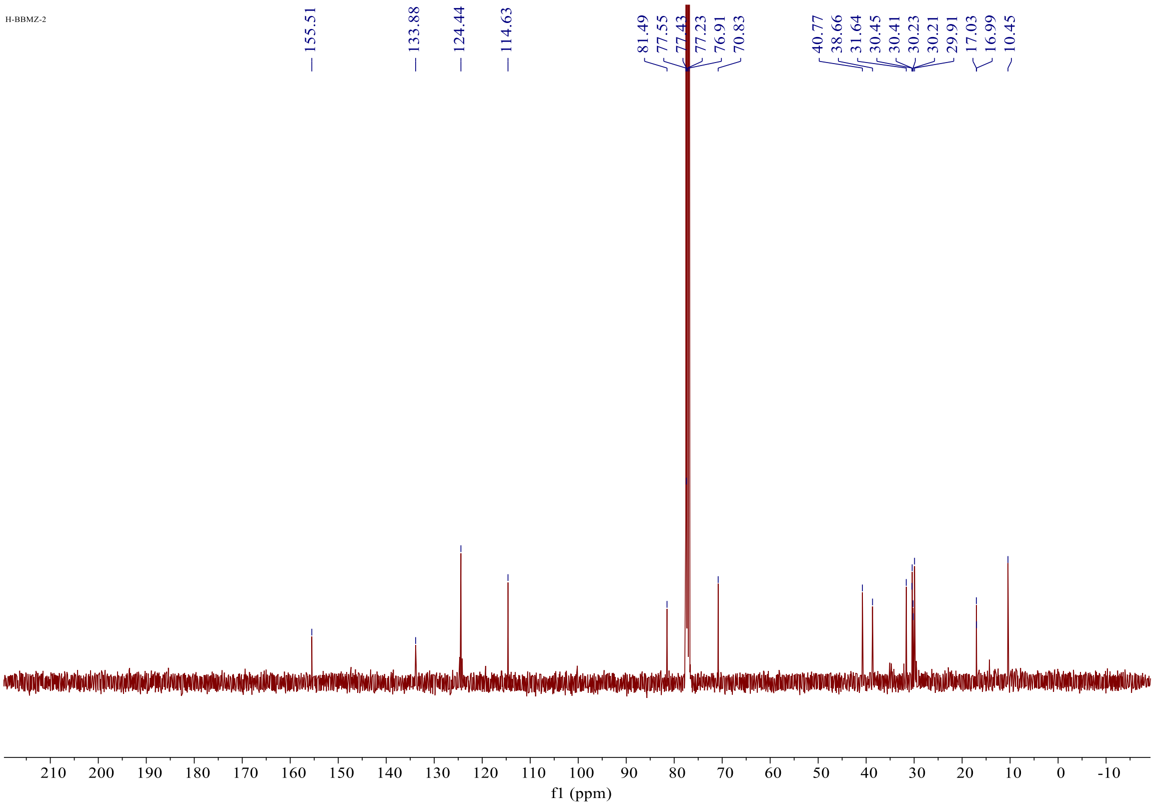

Figure S51. ^{13}C NMR spectrum of **21** in $CDCl_3$

© 2019 ACG Publications. All rights reserved.
